# Cadmium and glyphosate jointly compromise sperm function, fertilization, and early development in *Prochilodus magdalenae*

**DOI:** 10.3389/ftox.2025.1698489

**Published:** 2025-11-24

**Authors:** Jose Espinosa-Araujo, Lucellys Sierra-Marquez, Victor Atencio-Garcia, Jesus Olivero-Verbel

**Affiliations:** 1 Environmental and Computational Chemistry Group, School of Pharmaceutical Sciences. Zaragocilla Campus, University of Cartagena, Cartagena, Colombia; 2 Institute of Fishculture Research - CINPIC, University of Cordoba, Monteria, Colombia

**Keywords:** cadmium, glyphosate, sperm motility, mitochondrial integrity, fertilization, endocrine disruption, Prochilodus magdalenae

## Abstract

Cadmium (Cd) and glyphosate (Gly) are widespread aquatic contaminants known to impair reproductive function in freshwater fish. This study evaluated the effects of Cd, Gly, and their combined exposure on sperm quality and fertilization success in *Prochilodus magdalenae*, a commercially and ecologically important Neotropical species. Adult males were exposed to environmentally relevant concentrations of Cd, Gly, and a Cd + Gly mixture. Sperm motility parameters, membrane and mitochondrial integrity, DNA fragmentation, fertilization rate, and hatching success were assessed. Cd exposure significantly reduced sperm motility at 25 mg/L (45.0%), while Gly induced motility impairment at concentrations above 10 mg/L. Co-exposure to Cd and Gly further exacerbated the decline in motility (*p* < 0.05). Cd also caused significant mitochondrial and membrane damage at 25 mg/L, whereas Gly produced moderate alterations (*p* < 0.05). Fertilization rates declined across all treatments, with complete inhibition (0.0%) observed at the highest combined concentrations (Cd 25 mg/L + Gly 40 mg/L). No significant differences were found in sperm DNA fragmentation. This study shows that combined exposure to cadmium and glyphosate has a stronger negative impact on fish sperm quality than individual exposure. The effects depend on concentration, involving oxidative stress and alterations in motility and membrane integrity. These results contribute to understanding how mixed contaminants affect fish reproduction and emphasize the need for long-term studies under realistic environmental conditions.

## 1 Introduction

In Colombian aquatic systems, environmental pollution is an increasing concern with direct effects on the reproduction of native species. Among the most relevant contaminants are cadmium (Cd), mainly derived from mining and industrial activities, and glyphosate (Gly), a widely used herbicide in the country’s agricultural areas. Both act as endocrine disruptors, compromising essential reproductive parameters in Neotropical fish. Cd, a heavy metal commonly found in industrial and agricultural effluents, is highly toxic and bioaccumulates in fish tissues, leading to oxidative stress and reproductive dysfunction, including both structural and functional alterations in spermatozoa (Ali et al., 2022). Gly, a widely used herbicide in global agriculture, is frequently detected in water bodies near agricultural zones. Recent studies have shown that Gly and its commercial formulations (e.g., Roundup®) can induce endocrine disruption and impair sperm quality, affecting motility, DNA integrity, and fertilization capacity in fish and other aquatic vertebrates (Silveira et al., 2019; Fiorino et al., 2018; Yang et al., 2022).

Several studies have shown that both Cd and Gly disrupt key reproductive parameters in fish. Cd accumulates in the gonads and induces oxidative stress, reducing sperm motility and viability (Antar et al., 2023; Topal et al., 2015). From an ecotoxicological perspective, chronic Cd exposure can significantly decrease fertilization success and population viability by interfering with fundamental cellular processes essential for fertilization and embryonic development (Gárriz and Miranda, 2020). Although Gly was long considered a low-toxicity herbicide in vertebrates, solid evidence now demonstrates that it also interferes with spermatogenesis and fertilization in fish of commercial and ecological importance. (Le Du-Carrée et al., 2021).

The combined presence of Cd and Gly in aquatic ecosystems creates a complex scenario of multiple stressors, in which co-exposure can intensify toxic effects and accelerate reproductive dysfunction in fish (Miranda and Somoza, 2022). Simultaneous exposure has been shown to reduce sperm motility, viability, and fertilization success in various species. For instance, studies in zebrafish (*Danio rerio*) have shown that sublethal concentrations of Cd (0.5–10 μg/L) significantly reduce sperm quality and fertilization success (Acosta et al., 2016). In the case of Gly, Fiorino et al. (2018), used concentrations ranging from 0.005 to 50 mg/L to assess sublethal effects such as hatching rate and morphological abnormalities in embryos, although no specific values for reproductive endpoints in fish sperm were reported. Chronic exposure can also impair embryo hatching, with critical implications for ecosystem stability. (Socha et al., 2025). Despite their frequent co-occurrence in aquatic systems, the combined effects of Cd and Gly are still poorly understood, especially with respect to their potential additive, synergistic, or antagonistic interactions (Ramakrishnan et al., 2011; Kováčik et al., 2020).

*Prochilodus magdalenae* is a keystone species in Colombia’s inland fisheries, yet its population has declined by approximately 80% over the past 4 decades. In the Magdalena River basin, annual landings have dropped from an estimated 38,000 tons in 1978 to just 7,962.4 tons in 2022 (SEPEC, 2022). This decline poses serious socio-environmental consequences, as many small-scale fishing communities rely on healthy aquatic ecosystems for their livelihoods, which are increasingly threatened by competing economic activities (FAO, 2014). Factors contributing to the collapse of *Prochilodus magdalenae* populations include habitat degradation from organic and inorganic pollutants, wetland loss, hydroelectric dams, and overfishing (Montes-Petro, 2018; Landínez-García et al., 2020; Sidlauskas and Valderrama, 2022).

Given the documented presence of contaminants such as Cd and Gly in Colombian aquatic ecosystems, the selection of *P. magdalenae* as a native model species offers ecological and regional relevance for studying sublethal toxicological effects in freshwater fish. Reported environmental concentrations of Cd and Gly in Colombian freshwater systems support the ecological relevance of the exposure levels used in this study. For example, Cd levels in sediments from the Magdalena River have been reported up to 3.9 mg/kg (Tejeda-Benítez et al., 2018), while Gly concentrations as high as 2.8 mg/L have been detected in surface waters from the Boyacá region (Alza-Camacho et al., 2016). More recently, concentrations between 204 and 216 μg/L were reported in drinking water supplied from the Pamplonita and Zulia river sources in Norte de Santander (Álvarez-Bayona et al., 2022).

This study investigates the combined effects of these two pollutants on sperm quality and fertilization in *P. magdalenae*, with the aim of providing critical data to guide contaminant management and aquatic conservation strategies. Specifically, we evaluate changes in sperm motility, viability, and fertilization success under various exposure levels, contributing to a broader understanding of the ecological risks associated with these contaminants and supporting efforts to protect vulnerable aquatic habitats.

## 2 Materials and methods

### 2.1 Chemicals

Cadmium chloride (CdCl_2_, 99.99%, CAS 10108-64-2) and glyphosate [N-(phosphonomethyl) glycine, 98.0%, CAS 1071-83-6] were obtained from Sigma-Aldrich®. Milli-Q water (resistivity 18.2 MΩ cm at 25 °C) was used as the control medium for sperm quality assessments in all experiments.

### 2.2 Gamete collection

This study was conducted at the Institute for Fishculture Research (CINPIC) of the University of Córdoba (Montería, Colombia). Male and female *P. magdalenae* (aged >3 years) were maintained in earthen ponds. The average body weight of males (n = 10) and females (n = 3) was 310.5 ± 20.5 g and 435.4 ± 34.8 g, respectively. Gametes were obtained via hormonal induction using common carp pituitary extract, following Atencio-García et al. (2021). Gametes were extracted by applying gentle abdominal pressure in a cranio-caudal direction. Semen was collected into sterile, dry Eppendorf tubes after cleansing the genital papilla to avoid contamination from water, urine, or feces (Atencio-García et al., 2015).

### 2.3 Experimental conditions

The Gly concentrations were selected based on preliminary trials, which determined a NOAEL (No Observed Adverse Effect Level) below 2 mg/L. The Cd concentrations included the maximum permissible level for drinking water in Colombia (0.003 mg/L) (MESD, 2007). Five nominals Cd concentrations (0.0025, 0.025, 0.25, 2.5 and 25 mg/L) and five Gly concentrations (2.5, 5, 10, 20, and 40 mg/L) were assessed. Additionally, a control solution without contaminants was prepared.

For combination toxicity experiments, semen and embryos were exposed to binary mixtures of Cd and Gly following a completely randomized design. The treatments consisted of three Cd concentrations (0.0025, 0.25, and 25 mg/L) combined with three Gly concentrations (2.5, 10, and 40 mg/L), plus a control without contaminants. All treatments were evaluated in triplicate, and all experiments were repeated thrice.

The collected sperm was maintained in Eppendorf tubes wrapped in aluminum foil at a temperature between 27.0 ± 1.0 until its analysis in the CASA system (Microptic, SCA, Spain). The embryos were maintained in cylindrical-conical incubators with aged tap water and supplemental aeration at a temperature of 26.5 °C–29 °C, pH 6.0–7.1, dissolved oxygen above 7.0 mg/L, total ammonia below 0.1 mg/L, and a 12/12 h light/dark regime (Atencio-García et al., 2015).

### 2.4 Sperm quality assay

An aliquot of 0.25 µL of semen was activated with 75 µL of Milli-Q water previously contaminated with each contaminant (1:300 dilution). Sperm motility parameters were assessed using a computer assisted sperm analysis (CASA; Microptic SCA, Spain). The analytical setup included specification of frame rate (50 Hz), capture of 25 consecutive frames per assay, and a minimum sperm size threshold of 10 µm to avoid tracking artifacts. Motility was classified as rapid (>100 μm/s), medium (46–100 μm/s), or slow (10–45 μm/s), while cells with velocities <10 μm/s were considered immotile (Espinosa-Araujo et al., 2025). Total progressive motility, curvilinear velocity (VCL), and straight-line velocity (VSL) were recorded. Measurements were conducted over a 15 s period, starting 5 s post-activation and ending at 20 s. Activation time duration was defined as the time until approximately 90% of sperm ceased movement (Espinosa-Araujo et al., 2025).

### 2.5 Sperm membrane and mitochondrial damage

A 1 mL semen sample was incubated for 1 h in a medium containing 6% glucose and the corresponding concentrations of Cd, Gly, or their mixtures. The glucose solution maintained osmotic balance and prevented sperm activation (Martínez et al., 2011). After incubation, 1 µL of semen was mixed with 1 mL of a staining solution containing 3,3′-dihexyloxacarbocyanine iodide (DiOC6(3), 70 nM) and propidium iodide (PI, 2 μg/mL), and incubated in the dark for 20 min prior to flow cytometry evaluation. DiOC6(3) was used to assess mitochondrial integrity, while PI detected sperm membrane damage (Atencio-García et al., 2023).

Flow cytometry assays were conducted on an LSRFortessa™ flow cytometer (BD Biosciences, Franklin Lakes, NJ, USA) equipped with a 488 nm blue laser. DiOC6(3) fluorescence was detected using a 530/30 nm band-pass filter, while PI was collected with a 610/30 nm band-pass filter. Detector voltages were optimized using unstained controls, and fluorescence compensation was applied based on single-stain samples to correct spectral overlap. A minimum of 10,000 events was acquired per sample. Spermatozoa were identified by FSC vs. SSC characteristics, excluding debris and non-sperm particles, while doublets were removed using FSC-H vs. FSC-A plots. DNA fragmentation was analyzed using PI fluorescence, applying thresholds defined from negative controls. Chicken erythrocytes and sheep T cells from the BD DNA QC kit were used as internal standards. Data were acquired with FACSDiva software (Windows 7, version 6.1) and further analyzed with FlowJo v10.9.

### 2.6 Sperm DNA fragmentation

From each treatment, 1 µL of 6% glucose-incubated semen was fixed in 3 mL of 70% ethanol and stored at 20 °C for at least 12 h. Samples were then washed with ultrafiltered calcium-free PBS by vortex agitation and centrifuged at 2500 rpm for 10 min at 4 °C. After discarding the supernatant, the pellet was resuspended in 300 µL of PI solution with RNase A (20 U per million cells). Samples were mixed by vortex and incubated in the dark at room temperature for 20 min before flow cytometric analysis (Atencio et al., 2013).

### 2.7 Fertilization capacity assay

The effects of Cd, Gly, and their combinations on fertilization capacity (fertility and hatching rates), embryonic development, and hatching were evaluated by fertilizing 1 g of oocytes with 50 µL of sperm. Then, gametes were activated with 100 mL of contaminated water from each treatment and maintained for 1 h until the oocytes were fully hydrated (Sierra-Márquez et al., 2019). After hydration, the embryos were transferred to 2.5 L cylindrical-conical experimental incubators with an upward water constant flow system.

Fertility rate was estimated 6 hours post-fertilization, while hatching was evaluated 11 hours post-fertilization, when embryos were nearing hatching and exhibited peristaltic movements. Viable embryos were identified as transparent and at the expected developmental stage, whereas non-viable embryos were classified as opaque or exhibiting tissue detachment (Madariaga-Mendoza et al., 2023). The embryonic viability was assessed using a stereomicroscope (Carl Zeiss, Stemi-2000C, Germany) and an image analyzer (Carl Zeiss, Axiovision 4.8, Germany). The fertility and hatching rates were calculated using the following equations:

$$\text{Fertility }\left(\%\right)=\left(N{}^{\circ}\text{ viable oocytes}/N{}^{\circ}\text{ total oocytes}\right)\;\times \;100$$

$$\text{Hatching }\left(\%\right)=\left(N{}^{\circ}\text{ viable embryos}/N{}^{\circ}\text{ total embryos}\right)\;\times \;100$$

### 2.8 Statistical analysis

A completely randomized design was used to evaluate the effects of water contaminated with Cd, Gly, and their combinations on sperm and embryo quality in *P. magdalenae*. Normality and homogeneity of variances were verified using the Kolmogorov–Smirnov and Bartlett tests, respectively. Differences between means for variables across more than two groups were evaluated using ANOVA, and Dunnett’s test was applied to compare each sample with the control group. In the joint toxicity test, the assessed variables were analyzed using a two-way ANOVA to determine any interaction between Cd and Gly, creating a 3 × 3 ANOVA matrix. In all cases, *p <* 0.05 was considered statistically significant. All results were expressed as the mean ± standard error.

## 3 Results

### 3.1 Nominal concentration

#### 3.1.1 Sperm kinematics and fertilizing capacity

**FIGURE 1:**
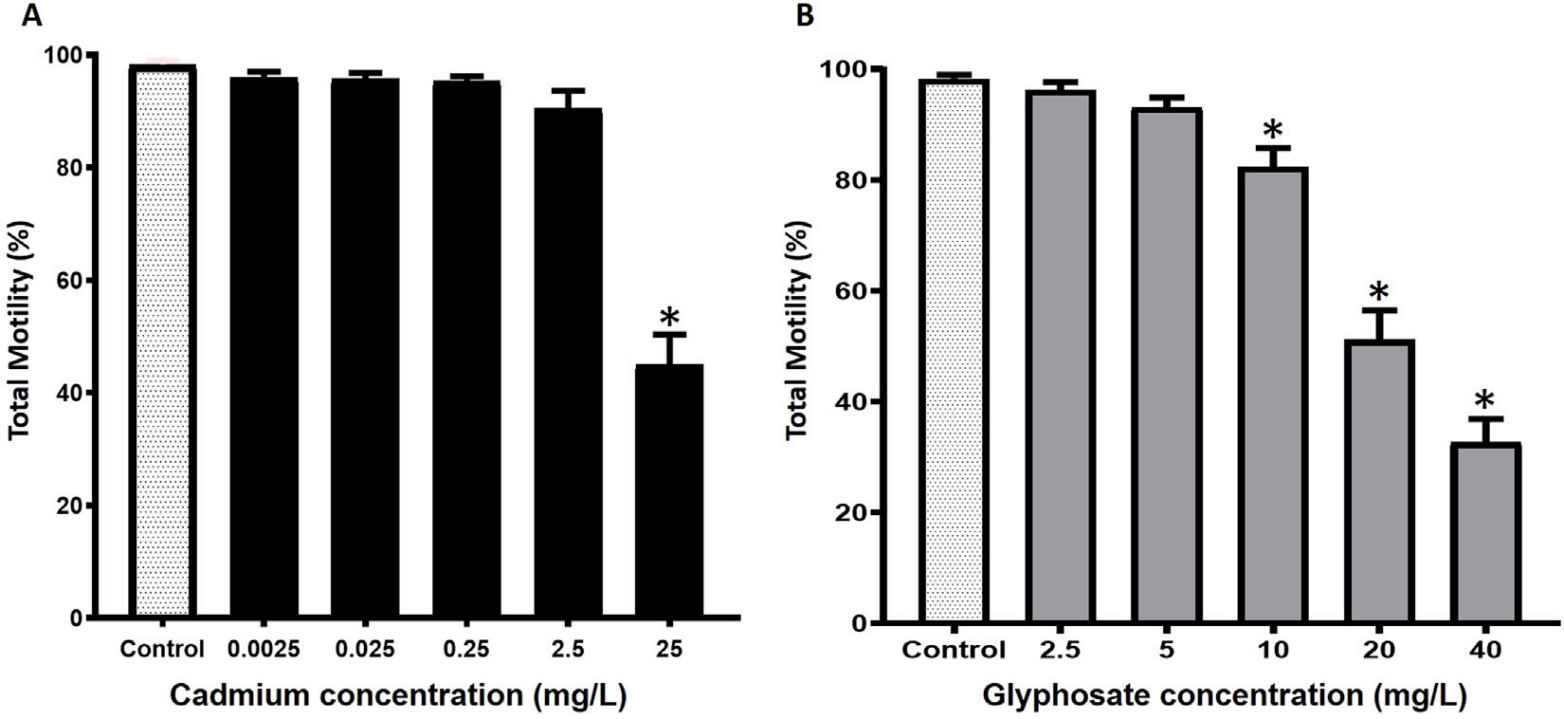
Total motility of *Prochilodus magdalenae* semen exposed to Cd **(A)** or Gly **(B)**. Values are expressed as mean ± standard error (SE). Asterisks indicate significant differences compared to the control group (*p* < 0.05).

The effects of Cd or Gly on total motility of *P. magdalenae* semen are shown in Figure 1. Total motility of Cd exposure semen did not show significant differences in the treatments with concentrations lower at 2.5 mg/L, concerning the control group (*p >* 0.05). However, at Cd 25 mg/L, sperm motility drastically decreased to 44.9% ± 5.4% (*p <* 0.05) (Figure 1A). In the case of Gly exposure semen, the lower concentrations (2.5 and 5 mg/L) did not show significant differences (*p >* 0.05), with sperm motility values of 96.3% ± 1.4% and 93.2% ± 1.7%, respectively. However, at 10 mg/L, motility decreased to 82.4% ± 3.4%, showing a significant difference compared to the control, a trend that persisted at 20 and 40 mg/L (*p* < 0.05) (Figure 1B).

The values presented in Table 1 correspond to sperm kinematic parameters in *P. magdalenae* semen exposed to Cd or Gly. The control group exhibited the highest values in all evaluated parameters, such as rapid spermatozoa (75% ± 4.4%), static (1.6% ± 0.7%), VCL (143.8 ± 22.2 μm/s), VSL (63.9 ± 5.0 μm/s), and a motility duration of 31.3 ± 0.2 s.

**TABLE 1:** Sperm kinematics parameters in *Prochilodus magdalenae* semen exposed to Cd or Gly. Values are expressed as mean ± standard error (SE). Asterisks indicate significant differences compared to the control (*p <* 0.05). VCL: Curvilinear velocity; VSL: Straight-line velocity.

Treatment (mg/L)	Parameters						
	Rapid (%)	Medium (%)	Slow (%)	Static (%)	VCL (μm/s)	VSL (μm/s)	Motility duration (s)
Control	75.0 ± 4.4	16.3 ± 3.7	7.0 ± 1.0	1.6 ± 0.7	143.8 ± 22.2	63.9 ± 5.0	31.3 ± 0.2
Cd 0.0025	67.6 ± 5.6	19.6 ± 4.1	8.6 ± 1.6	4.2 ± 1.2	122.2 ± 6.9	59.4 ± 3.0	32.5 ± 0.2
Cd 0.025	63.2 ± 5.6	21.1 ± 4.7	11.4 ± 1.3	4.3 ± 1.1	122.8 ± 8.0	58.3 ± 3.4	30.6 ± 1.6
Cd 0.25	59.2 ± 6.6	27.7 ± 6.0	11.1 ± 1.5	4.7 ± 0.9	112.8 ± 8.3	57.8 ± 4.3	29.0 ± 1.3
Cd 2.5	52.7 ± 7.2*	19.1 ± 3.8	17.6 ± 3.4*	9.6 ± 3.2	125.9 ± 22.7	62.6 ± 6.4	23.9 ± 2.6*
Cd 25	6.8 ± 1.8*	11.5 ± 2.7	31.9 ± 2.5*	49.9 ± 5.5*	42.8 ± 6.5*	26.1 ± 5.5*	16.1 ± 2.2*
Gly 2.5	63.7 ± 7.3	22.5 ± 4.6	10.0 ± 2.1	3.7 ± 1.4	114.5 ± 7.8	65.5 ± 8.3	31.8 ± 1.6
Gly 5	53.2 ± 6.8*	26.4 ± 4.9	13.6 ± 2.2	6.8 ± 1.7	106.9 ± 8.1	55.4 ± 1.7	30.0 ± 1.3
Gly 10	37.5 ± 7.0*	22.4 ± 3.2	22.5 ± 3.4*	17.6 ± 3.4*	86.7 ± 8.9*	47.4 ± 3.1	28.5 ± 0.7
Gly 20	9.6 ± 2.1*	13.6 ± 3.0	28.0 ± 1.4*	48.8 ± 5.3*	50.1 ± 5.0*	22.6 ± 3.2*	21.3 ± 1.0*
Gly 40	1.3 ± 0.5*	5.8 ± 2.3	25.6 ± 1.5*	67.3 ± 4.2*	30.1 ± 2.9*	9.4 ± 3.0*	5.8 ± 2.9*

Semen exposed to Cd concentrations between 0.0025 and 0.25 mg/L showed no significant differences in sperm kinematic parameters with the control group values (*p* > 0.05). In Cd 2.5 mg/L, the rapid sperm percentage was 52.7% ± 7.2%, with an increase in the slow spermatozoa (17.6% ± 3.4%) and decreasing motility duration of 23.9 ± 2.6 s (*p <* 0.05). In Cd 25 mg/L, rapid motility (6.8% ± 1.8%), VCL (42.8 ± 6.5 μm/s), VSL (26.1 ± 5.5 μm/s) and motility duration (16.1 ± 2.2 s) reached its lowest values and the highest static spermatozoa rate (49.9% ± 5.5%) (*p* < 0.05).

Semen exposed to Gly 2.5 mg/L showed no significant difference with the sperm kinematic values of the control group (*p* < 0.05). At the highest Gly concentration (40 mg/L), rapid motility decreased to 1.3% ± 0.5%, with 67.3% ± 4.2% static spermatozoa, VCL of 30.1 ± 2.9 μm/s, VSL of 9.4 ± 3.0 μm/s, and a motility duration of 5.8 ± 2.9 s (*p <* 0.05).

**FIGURE 2:**
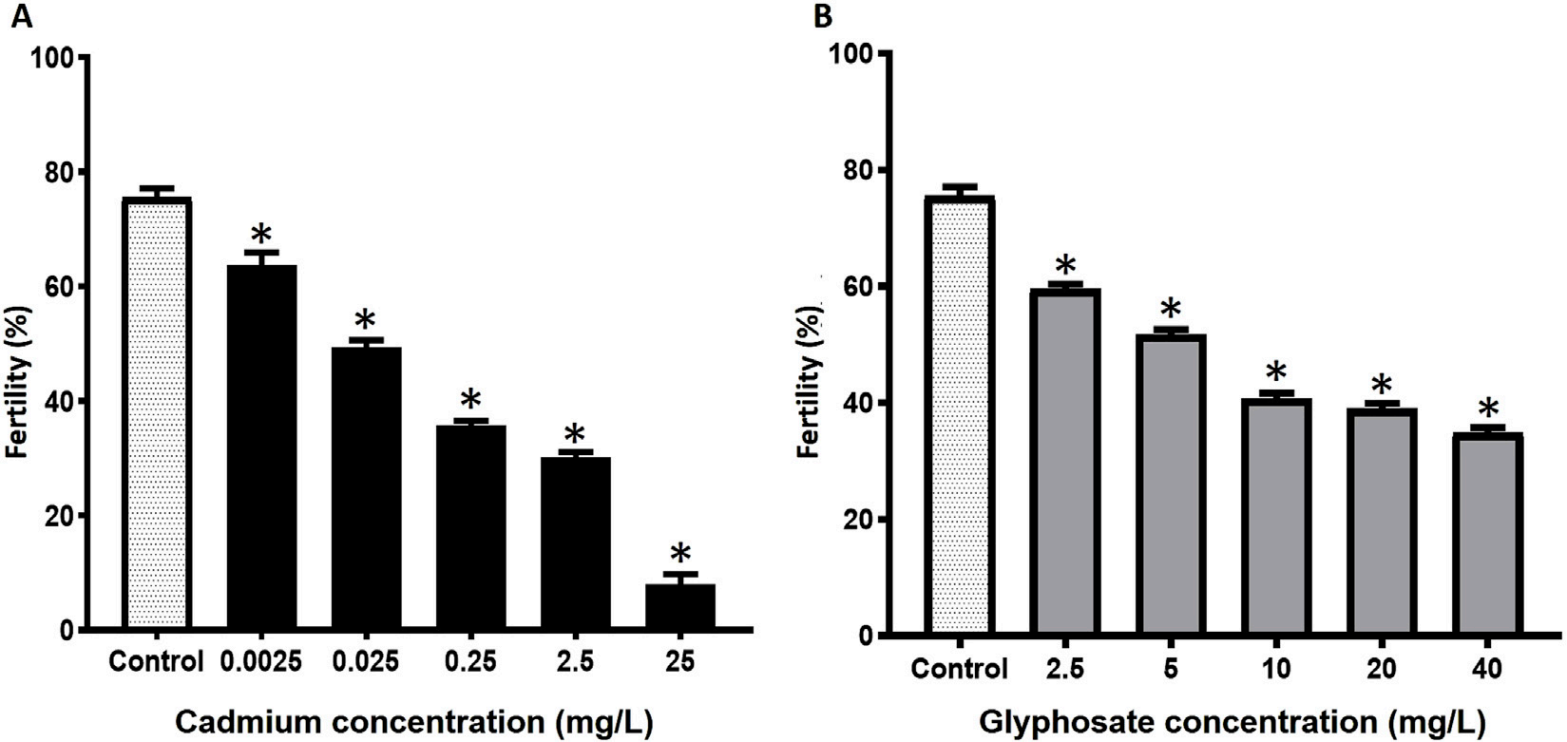
Fertility rates in *Prochilodus magdalenae* oocytes exposed to Cd **(A)** or Gly **(B)**. Values are expressed as mean ± standard error (SE). Asterisks indicate significant differences compared to the control group (*p* < 0.05).

The fertility rates in *P. magdalenae* oocytes exposed to Cd or Gly are shown in Figure 2. Exposure to Cd caused significant reductions compared to the control group (p < 0.05), with fertility progressively decreasing from 63.7% ± 2.2% (0.0025 mg/L) to 8.0% ± 1.7% (25 mg/L) (Figure 2A). In the Gly exposure treatments, significant reductions were also observed in all cases, with fertility values ranging from 34.9% ± 0.8% (40 mg/L) to 59.5% ± 0.9% (2.5 mg/L) (Figure 2B).

**FIGURE 3:**
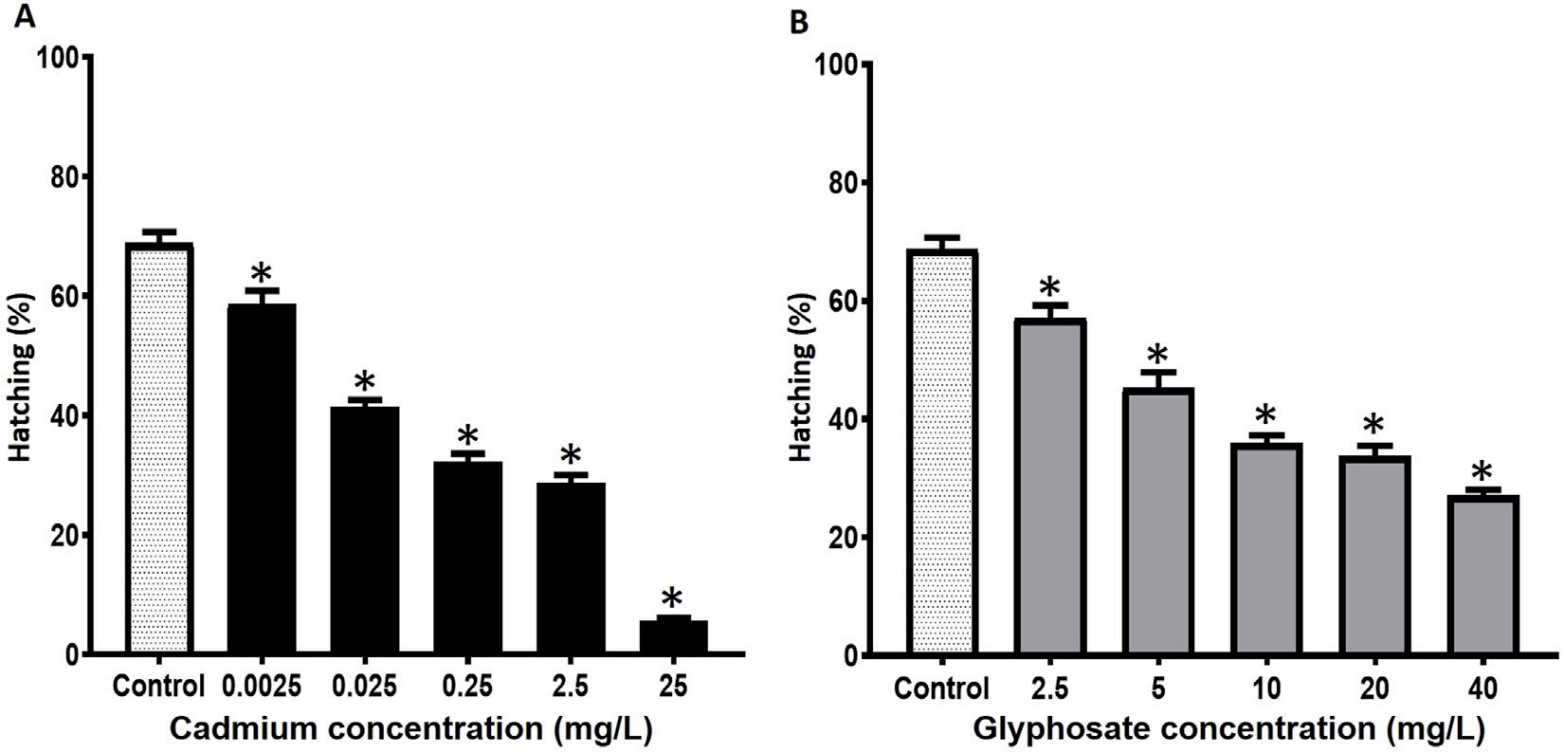
Hatching rates in *Prochilodus magdalenae* oocytes exposed to Cd **(A)** or Gly **(B)**. Values are expressed as mean ± standard error (SE). Asterisks indicate significant differences compared to the control group (*p* < 0.05).

The hatching rates in *P. magdalenae* gametes exposed to Cd or Gly are shown in Figure 3. The hatching of the gametes exposed to the different concentrations of Cd analyzed showed statistical differences with the value of the control group (*p* < 0.05), ranging from 58.7% ± 2.2% (0.0025 mg/L) to 5.7% ± 0.4% (25 mg/L) (*p* < 0.05) (Figure 3A).

Regarding Gly exposure, all analyzed concentrations showed a significant reduction in hatching rate compared to the control group, ranging from 57.2% ± 2.0% (2.5 mg/L) to 27.1% ± 1.0% (40 mg/L) (*p* < 0.05) (Figure 3B).

#### 3.1.2 Mitochondrial, membrane, and DNA damage.

**FIGURE 4:**
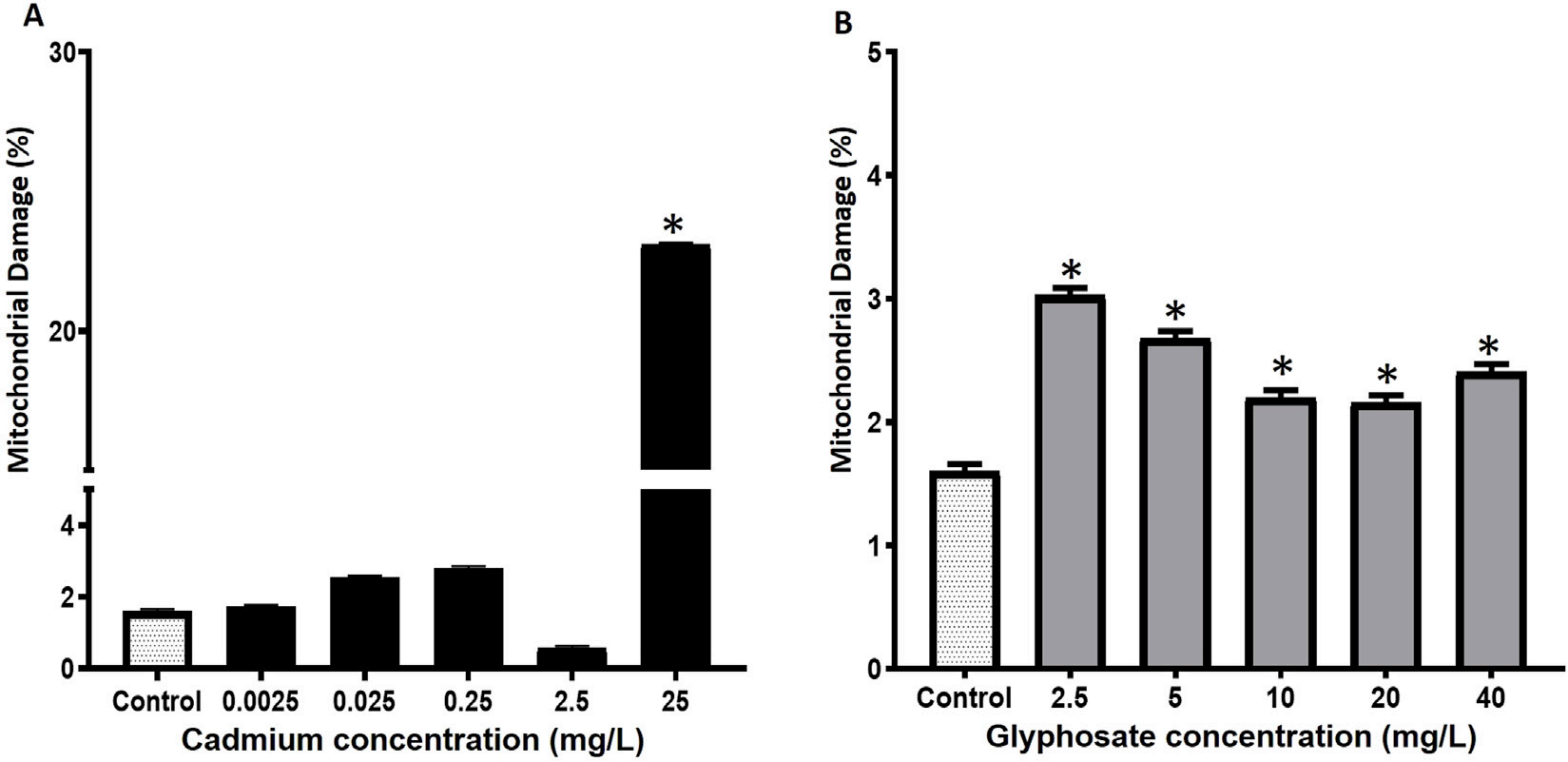
Mitochondrial damage percentages in *Prochilodus magdalenae* semen exposed to Cd **(A)** or Gly **(B)**. Values are expressed as mean ± standard error (SE). Asterisks indicate significant differences compared to the control group (*p* < 0.05).

The mitochondrial damage percentages in *P. magdalenae* semen exposed to Cd or Gly are shown in Figure 4. Cd exposure did not induce significant alterations in sperm mitochondria at concentrations between 0.0025 and 2.5 mg/L (p > 0.05), showing a response comparable to the control group. However, at 25 mg/L (23.1% ± 0.1%), a significant increase in mitochondrial damage was observed (p < 0.05).

For Gly exposure alone, a significant increase in mitochondrial damage was observed at all evaluated concentrations compared to the control, with values ranging from 2.2% ± 0.1% to 3.0% ± 0.1% (*p* < 0.05) (Figure 4B).

**FIGURE 5:**
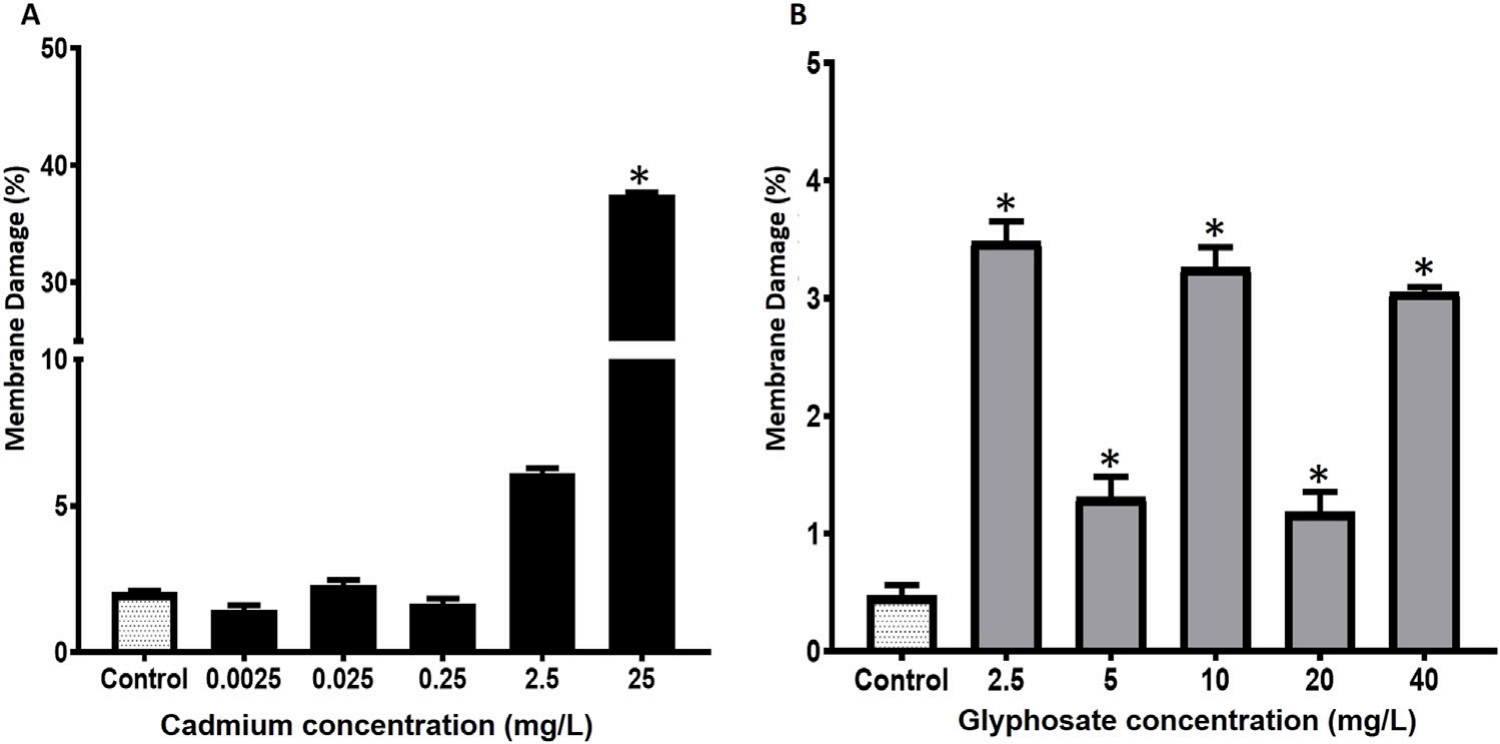
Membrane damage percentages in *Prochilodus magdalenae* semen exposed to Cd **(A)** or Gly **(B)**. Values are expressed as mean ± standard error (SE). Asterisks indicate significant differences compared to the control group (*p* < 0.05).

The sperm membrane damage percentages in *P. magdalenae* semen exposed to Cd or Gly are shown in Figure 5. Cd exposure did not cause significant differences in sperm membrane damage at concentrations between 0.0025 and 0.25 mg/L compared to the control (p > 0.05). However, at 2.5 mg/L (6.1% ± 0.1%) and 25 mg/L (37.5% ± 0.1%), membrane damage increased significantly compared to the control (p < 0.05).

Gly exposure caused a significant increase in sperm membrane damage at all evaluated concentrations compared to the control, ranging from 1.2% ± 0.1% (20 mg/L) to 3.5% ± 0.1% (2.5 mg/L) (*p* < 0.05) (Figure 5B).

**FIGURE 6:**
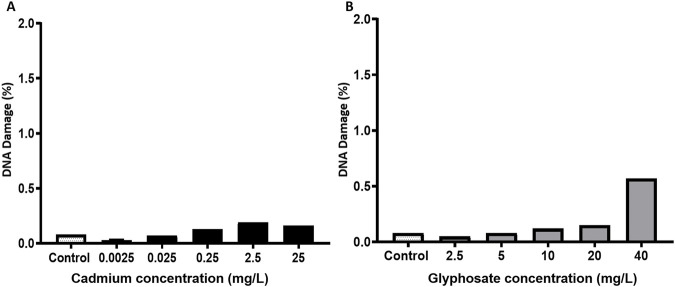
DNA damage percentages in *Prochilodus magdalenae* semen exposed to Cd **(A)** or Gly **(B)**. Values are expressed as mean ± standard error (SE). Asterisks indicate significant differences compared to the control group (*p* < 0.05).

The DNA damage percentages in *P. magdalenae* semen exposed to Cd or Gly are shown in Figure 6. No significant differences in sperm DNA damage were observed in *P. magdalenae* at any of the evaluated concentrations of Cd or Gly (*p >* 0.05).

### 3.2 Mixtures

#### 3.2.1 Sperm kinematics and fertilizing capacity

In Table 2, the sperm kinematic parameters of *P. magdalenae* under combined Cd and Gly exposure are shown. For the combined treatments, a reduction in sperm kinematics was observed in all cases, except for the Cd 0.0025+Gly 2.5 mg/L combination, where fast sperm (51.6% ± 8.8%), medium sperm (28.6% ± 7.2%), VSL (52.0 ± 6.1 μm/s), and motility duration (29.6 ± 0.7 s) were recorded, with no significant differences compared to the control (*p* > 0.05). Detailed sperm motility parameters, including rapid, medium, slow, and static categories, as well as velocity indices, are shown in Supplementary Figures S1–S7.

**TABLE 2:** Sperm kinematic parameters in *Prochilodus magdalenae* semen exposed to combined Cd and Gly treatments. Values are expressed as mean ± standard error (SE). Asterisks indicate significant differences compared to the control (*p <* 0.05). VCL: Curvilinear velocity; VSL: Straight-line velocity.

Treatment (mg/L)	Parameters						
	Rapid (%)	Medium (%)	Slow (%)	Statics (%)	VCL (μm/s)	VSL (μm/s)	Motility duration (s)
Control	75.0 ± 4.4	16.3 ± 3.7	7.0 ± 1.0	1.6 ± 0.7	143.8 ± 22.2	63.9 ± 5.0	31.3 ± 0.2
Cd 0.0025+Gly 2.5	51.6 ± 8.8	28.6 ± 7.2	15.4 ± 2.8*	4.5 ± 1.0	102.6 ± 8.5*	52.0 ± 6.1	29.6 ± 0.7
Cd 0.0025+Gly 10	48.7 ± 4.8*	20.8 ± 3.3	18.8 ± 1.7*	11.7 ± 1.6	103.4 ± 6.2*	51.5 ± 2.3	28.6 ± 0.7
Cd 0.0025+Gly 40	0.4 ± 0.2*	1.7 ± 0.4*	19.8 ± 1.5*	78.1 ± 1.8*	24.2 ± 1.5*	6.0 ± 1.1*	0.0 ± 0.0*
Cd 0.25+Gly 2.5	47.7 ± 9.5*	29.4 ± 6.1	16.0 ± 3.4*	7.0 ± 1.5	93.0 ± 14.0*	48.8 ± 4.3	28.1 ± 2.2
Cd 0.25+Gly 10	39.5 ± 8.5*	25.0 ± 2.9	19.1 ± 2.5*	16.4 ± 4.3*	87.3 ± 9.0*	43.5 ± 4.5*	23.2 ± 2.1*
Cd 0.25+Gly 40	0.5 ± 0.3*	2.2 ± 0.2	18.0 ± 1.2*	79.3 ± 1.3*	26.6 ± 2.0*	5.5 ± 1.4*	0.0 ± 0.0*
Cd 25+Gly 2.5	39.0 ± 8.1*	18.8 ± 2.8	21.8 ± 2.3*	20.5 ± 5.6*	93.7 ± 10.2*	48.5 ± 6.0	22.5 ± 2.4*
Cd 25+Gly 10	21.8 ± 6.3*	9.6 ± 2.0	24.3 ± 1.3*	44.3 ± 5.4*	71.0 ± 9.2*	31.2 ± 4.9*	20.8 ± 2.6*
Cd 25+Gly 40	1.0 ± 0.5*	2.9 ± 0.9	21.4 ± 1.8*	74.6 ± 2.6*	28.4 ± 2.8*	5.9 ± 1.1*	0.0 ± 0.0*

**FIGURE 7:**
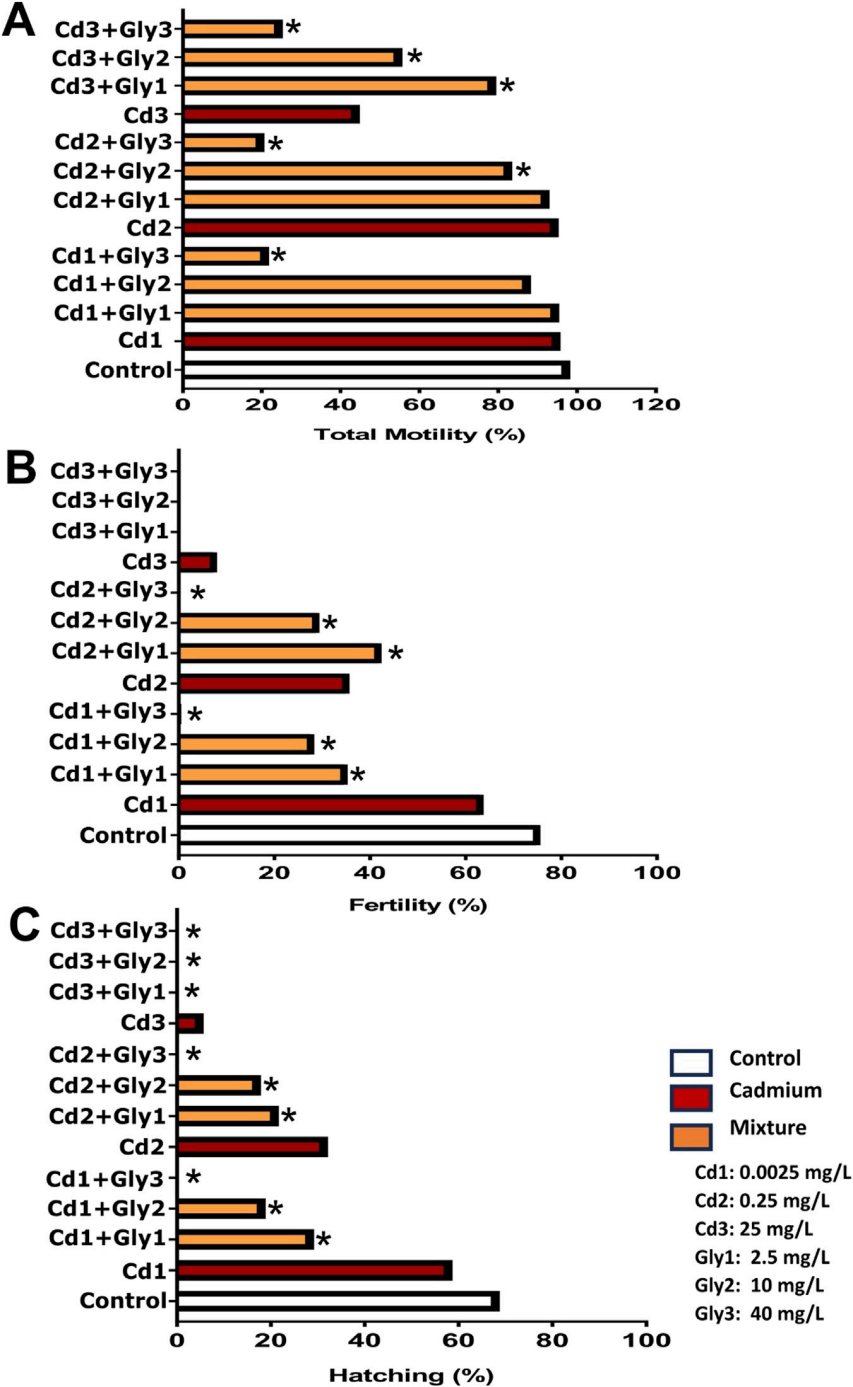
Effects of combined Cd–Gly exposures on total motility **(A)**, fertility **(B)**, and hatching **(C)** of *Prochilodus magdalenae*. Several Cd–Gly mixtures with their nominal concentrations are presented. Values are expressed as mean ± standard error (SE). Asterisks indicate significant differences of the mixtures compared to the control group (*p <* 0.05).

The total motility, fertility, and hatching rates of *P. magdalenae* semen exposed to combined Cd and Gly treatments are shown in Figure 7. For the Cd and Gly combination exposures, total motility was not statistically different from the control in Cd 0.0025+Gly 2.5 mg/L (95.6% ± 1.0%), Cd 0.0025+Gly 10 mg/L (88.26% ± 1.6%), and Cd 0.25+Gly 2.5 mg/L (93.0% ± 1.5%) (*p >* 0.05). However, for the remaining combinations, a considerable reduction in motility was observed (*p <* 0.05) (Figure 7A). Fertility showed even more pronounced reductions, with values ranging from 0.0% ± 0.0% (25 mg/L Cd+40 mg/L Gly) to 42.4% ± 2.1% (0.25 mg/L Cd+2.5 mg/L Gly), and all combinations differed significantly from the control group (Figure 7B). Hatching rates were also significantly reduced in all combinations compared to the control (*p <* 0.05), and no hatching was observed in Cd 0.0025+Gly 40 mg/L, Cd 0.25+Gly 40 mg/L, Cd 25+Gly 2.5 mg/L, Cd 25+Gly 10 mg/L, and Cd 25+Gly 40 mg/L treatments (Figure 7C). Representative micrographs of early embryonic development under Cd, Gly, and combined exposures are provided in Supplementary Figures S8–S10.

#### 3.2.2 Mitochondrial, membrane, and DNA damage

**FIGURE 8:**
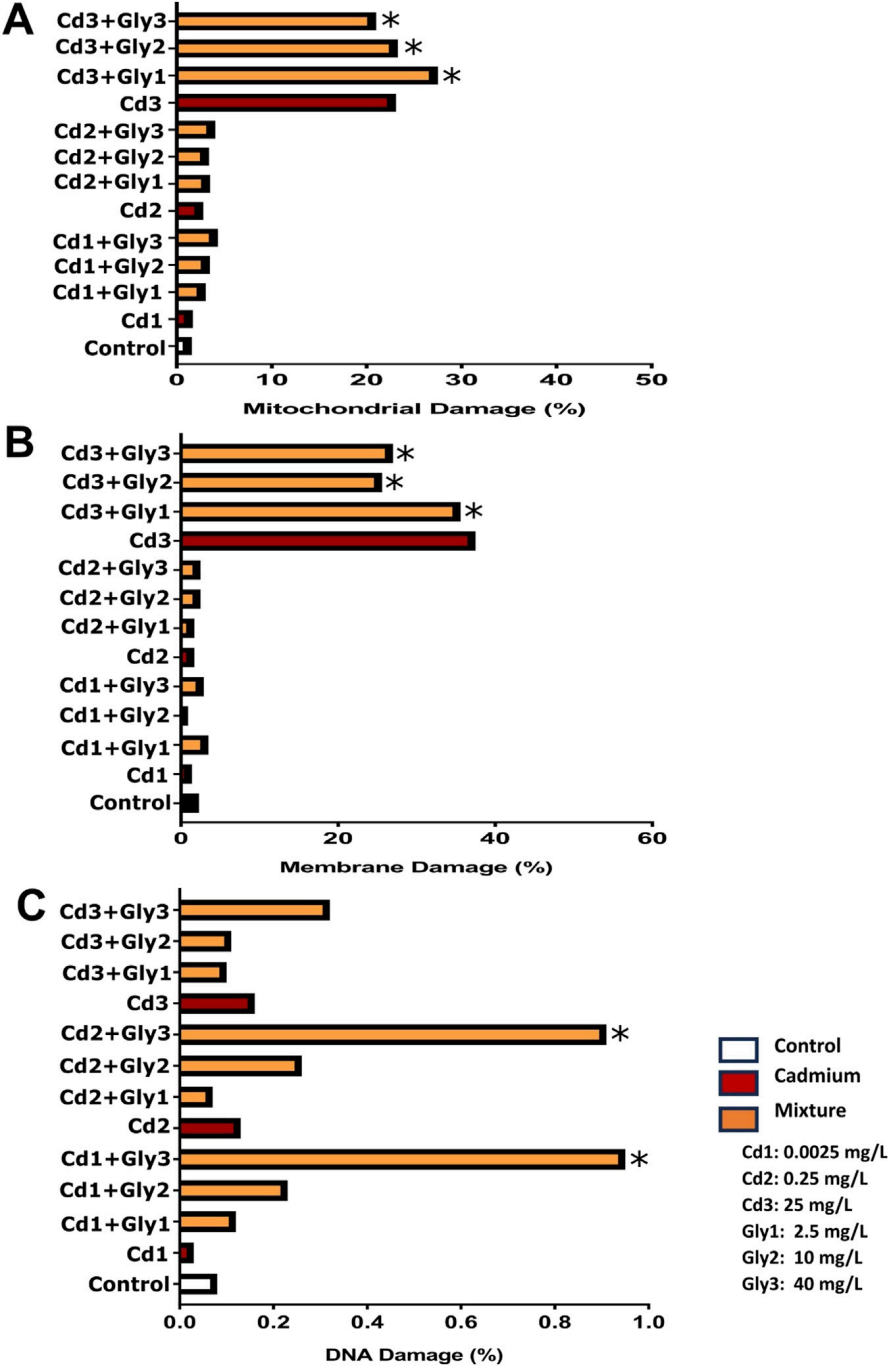
Effects of combined Cd–Gly exposures on mitochondrial damage **(A)**, membrane damage **(B)**, and DNA damage **(C)** in *Prochilodus magdalenae* semen. Several Cd–Gly mixtures with their nominal concentrations are presented. Values are expressed as mean ± standard error (SE). Asterisks indicate significant differences of the mixtures compared to the control group (*p <* 0.05).

The mitochondrial, membrane, and DNA damage in *P. magdalenae* semen exposed to combined Cd and Gly treatments are shown in Figure 8. In treatments combining Cd and Gly, a significant increase in mitochondrial damage was observed in all combinations, with values ranging from 3.06% ± 0.1% (0.0025 Cd + Gly 2.5) to 27.5% ± 0.1% (25 Cd + Gly 2.5) (*p <* 0.05) (Figure 8A). For membrane damage, significant differences compared to the control were found only in combinations that included Cd 25 mg/L (*p <* 0.05) (Figure 8B). With respect to sperm DNA integrity, significant differences were observed only in the combinations Cd 0.0025+Gly 40 mg/L and Cd 0.25+Gly 40 mg/L. No differences were detected for the other evaluated combinations compared with the control (*p >* 0.05) (Figure 8C).

## 4 Discussion

The widespread use of pesticides and heavy metals in agriculture has become a growing concern due to its implications for both environmental sustainability and human health (Cai et al., 2023; Cordeiro et al., 2023; Liu et al., 2022). These pollutants not only deteriorate soil and water quality but also accumulate along the food chain, raising serious questions about their potential health risks (Alengebawy et al., 2021; Pei et al., 2022; Wang et al., 2019). Moreover, recent evidence indicates that different contaminants may interact in complex ways, producing cumulative or even synergistic effects, sometimes at concentrations previously considered safe (Zhou et al., 2004).

The progressive decline in sperm kinematics observed with increasing Cd and Gly concentrations indicates that both contaminants disrupt key mechanisms involved in sperm motility. This impairment is consistent with previous evidence showing that Cd induces oxidative stress in germ cells, promoting lipid peroxidation, plasma membrane damage, and mitochondrial dysfunction, all of which compromise sperm performance (Gautam et al., 2024). At low Cd levels, however, the activation of cellular defense systems such as metallothionein synthesis and antioxidant enzyme activity may transiently mitigate toxicity. Nonetheless, at higher concentrations, these compensatory responses become insufficient, leading to marked losses in motility and potential impairment of DNA integrity and fertilization capacity (Jezierska and Witeska, 2001).

Sierra-Márquez et al. (2019) reported that Cd reduced motility types in *P. magdalenae* semen, decreasing both rapid (46.5%) and medium (16.4%) motility at 25 and 0.25 mg/L, respectively. Other studies reported reduced motility in *Danio rerio* at concentrations of 10 mg/L (Acosta et al., 2016) and in *Colossoma macropomum* at concentrations above 1 mg/L (Pinto et al., 2021). These results could suggest that the effects or responses to contaminant exposure vary by species.

At the lowest Gly concentrations tested (2.5 and 5 mg/L), total sperm motility remained high (96.3% and 93.2%), consistent with previous studies showing that very low levels of this herbicide may not produce clear alterations in sperm function (Nerozzi et al., 2020). In contrast, from 10 mg/L onwards, motility declined significantly (82.4%) and the reduction became more pronounced at 20 and 40 mg/L. These results point to the existence of a toxicity threshold, beyond which Gly begins to impair sperm motility parameters. Similar patterns have been reported in other species: Akça et al. (2021) observed reduced motility in *Oncorhynchus mykiss* semen at 10 mg/L (39.2%), while Santos et al. (2023) documented a 30% reduction in *C. macropomum* at just 6 mg/L compared with controls. These findings are in line with the progressive decline detected in the present study from 10 mg/L of Gly. Importantly, the mixed-exposure experiments revealed how the interaction between Cd and Gly modifies the individual toxic responses, highlighting the relevance of assessing their combined rather than isolated effects. In combined exposures, sperm motility was not statistically different from the control at Cd 0.0025 + Gly 2.5 mg/L (95.55%), Cd 0.0025 + Gly 10 mg/L (88.26%), and Cd 0.25 + Gly 2.5 mg/L (93.04%). However, the other mixtures caused a marked reduction in motility. Joint exposure to Cd and Gly therefore represents a greater risk to sperm viability than either contaminant alone. Alengebawy et al. (2021) reported that this combination exacerbates oxidative stress, promoting apoptosis and further reducing sperm viability, while Spinaci et al. (2020) noted that Gly can enhance Cd uptake in reproductive cells, amplifying its cytotoxic action and producing a concentration-dependent decline in sperm motility and viability.

In the present study, the reduction in rapid sperm motility from 75% in the control group to only 6.8% at 25 mg/L of Cd is consistent with previous research suggesting that chronic Cd exposure decreases sperm viability and increases the percentage of immobile cells. Sierra-Márquez et al. (2019) reported a significant reduction in the percentage of fast-moving sperm to less than 20% after exposure to 25 mg/L Cd, which is similar to the results obtained in this study. However, one of the main findings of this study was observed when Cd at 25 mg/L was combined with Gly at concentrations of 2.5 or 10 mg/L, showing a slight increase in the percentage of rapid motility compared with Cd alone at 25 mg/L. In our activation medium, low Gly levels (2.5–10 mg/L) may form Cd–Gly complexes that decrease the availability of free Cd^2+^ ions, temporarily reducing their interaction with sperm motility mechanisms. This could explain the small, non-significant increase in rapid motility compared with Cd alone. However, at higher Gly concentrations, this protective interaction is no longer effective, and the combined toxicity of both compounds becomes predominant.

Although this difference may not be statistically significant, it suggests that the presence of a low Gly concentration might somewhat attenuate the toxic effect of Cd at that level. Some studies have demonstrated that chemical compounds can interact in the organism in a synergistic or antagonistic manner. For instance, Martin (2023) discusses how different heavy metals and pesticides interact in the reproductive system, affecting sperm function and other cellular processes. Aguilar-Juárez et al. (2025) also reported synergistic effects between Cd and Hg mixtures, which produced greater sperm motility impairment in marine fish than either metal alone. These contrasting findings suggest that the type of interaction synergistic or antagonistic depends on the physicochemical nature of the compounds involved. In the present study, combined exposure reduced toxicity in rapid sperm percentage and velocity values when Cd was included at 25 mg/L and Gly between 2.5 and 10 mg/L, which is consistent with reports from other authors suggesting a chelating interaction between the two chemical substances (Zhou et al., 2014).

The interaction between Cd and Gly appears to depend on concentration, with the potential to shift between antagonistic and synergistic responses. Zhou et al. (2014) observed in *Eisenia fetida* that certain concentrations of Gly reduced Cd toxicity by decreasing mortality and metal accumulation, likely due to its amine, carboxylate, and phosphonate functional groups, which enable strong chelation of heavy metals. Similarly, Zhihang et al. (2024) reported a concentration-dependent interaction between Cd and Gly, with antagonistic effects at lower doses that mitigated phenotypic damage in *Caenorhabditis elegans*. A comparable trend was observed for some sperm motility variables in the present study. Overall, these findings demonstrate that the toxicological outcomes of Cd and Gly co-exposure cannot be fully predicted from their individual effects alone, confirming that mixture interactions play a central role in shaping the observed responses in *P. magdalenae*.

Significant reductions in fertility and hatching were observed in all Cd concentrations, with values ranging from 63.7% at the lowest concentration (0.0025 mg/L) to 8.0% at the highest (25 mg/L). These results are consistent with previous studies demonstrating that Cd is an endocrine disruptor that affects gonadal function and gamete viability (Gautam et al., 2024). Sierra-Márquez et al. (2019) reported that Cd induced a concentration-dependent effect on *P. magdalenae* fertilization, showing significant decreases in fertility and hatching rates even when female eggs were exposed to 0.0025 mg/L of Cd. Similarly, Pinto et al. (2021) reported significant reductions in fertility and hatching in *C. macropomum* oocytes at Cd concentrations ranging from 0.6 to 1.8 mg/L, an effect also observed in this study.

Gly treatments also produced clear reductions in fertility, with values ranging from 59.5% at 2.5 mg/L to 34.9% at 40 mg/L. In roosters, chronic exposure to Roundup®, a Gly-based herbicide, has been shown to alter the expression of genes involved in oogenesis and to compromise oocyte viability through increased oxidative stress and activation of apoptotic pathways (Serra et al., 2021). These mechanisms could partly explain the reduction in fertility observed in the present study, as oxidative and apoptotic damage may also impair gamete quality and embryonic development in fish. When Cd and Gly were applied together, fertility declined even further, from 42.4% at Cd 0.25 mg/L + Gly 2.5 mg/L down to 0.0% at Cd 25 mg/L + Gly 40 mg/L, indicating a synergistic effect that particularly impacts fertility and hatching success.

This study also demonstrates that Cd and Gly exposure induces mitochondrial damage in spermatozoa, with greater effects when administered in combination. No significant differences in mitochondrial damage were observed at Cd concentrations between 0.0025 mg/L (1.71%) and 2.5 mg/L (0.57%) compared with the control group. However, at the highest concentration (25 mg/L), mitochondrial damage significantly increased to 23.1%. Acosta et al. (2016) found that Cd exposure at concentrations between 5 and 10 ppb in D. rerio sperm affected mitochondrial structure. In contrast, mitochondrial effects in this study were only observed at Cd concentrations of 25 mg/L. Cd can affect mitochondrial membrane integrity, reducing the efficiency of the electron transport chain and increasing ROS release, leading to a significant decrease in motility and sperm viability, with alterations in oxidative phosphorylation (Hardneck et al., 2021). Cd accumulation in the testes and sperm also causes calcium overload in mitochondria, triggering apoptosis (Ali et al., 2022). Such apoptosis compromises sperm function by promoting structural and metabolic deterioration, which reduces motility, impairs membrane integrity, and limits the sperm’s ability to fertilize the oocyte.

Exposure to Gly caused a significant increase in mitochondrial damage at all evaluated concentrations, with values ranging from 1.6% to 27.5%. Lopes et al. (2014) reported in *D. rerio* that exposure to Gly reduced sperm motility and motility duration at 5–10 mg/L, and at 10 mg/L also decreased mitochondrial functionality and the integrity of the membrane and DNA. Similarly, Anifandis et al. (2017) observed that Roundup exposure at 1 mg/L decreased progressive motility and mitochondrial dye incorporation in human sperm, indicating mitochondrial dysfunction, while Anifandis et al. (2018) found that Gly alone at 0.36 mg/L reduced motility without increasing DNA fragmentation at 1 h. In *Odontesthes bonariensis*, recent work showed formulation-dependent effects of Gly on sperm quality and early life stages (Menéndez-Helman et al., 2025).

In treatments where Cd and Gly were combined, all tested mixtures produced a significant increase in mitochondrial damage compared with the control group, pointing to a potential synergistic interaction. Cd is known to disrupt calcium homeostasis and trigger apoptotic pathways, whereas Gly contributes to mitochondrial energy collapse. Together, these processes may intensify oxidative stress beyond the capacity of cellular defenses, thereby accelerating mitochondrial dysfunction (Gautam et al., 2024). This synergistic response suggests that the Cd–Gly combination enhances mitochondrial instability, leading to greater loss of membrane potential and reduced energy availability in spermatozoa.

Cd at high concentrations (2.5 and 25 mg/L) was the most decisive factor in altering sperm membrane integrity. Although Gly caused some differences compared with the control, the level of damage did not exceed 5%, suggesting a relatively minor impact. Cd concentrations lower than 0.25 mg/L did not show significant differences in membrane damage, but at 2.5 mg/L (6.1%) and 25 mg/L (37.5%), a significant increase was observed. Cd can oxidize membrane lipids and reduce fluidity. This effect has been observed in testicular cells and fish sperm, where the metal induces structural disorganization and disrupts cellular homeostasis (Hatef et al., 2013). It has been reported that Cd increases lipid peroxidation in the sperm membrane, compromising fluidity and reducing viability (Acosta et al., 2016). In *C. macropomum*, Cd exposure above 1.2 mg/L decreased sperm motility and viability, reduced fertilization capacity, and increased nuclear fragmentation, likely associated with membrane damage and oxidative stress (Pinto et al., 2021). These results are consistent with our findings in *P. magdalenae*, where Cd exposure also compromised sperm membrane integrity and fertility.

Exposure to Gly also increased membrane damage at all concentrations, although not above 5%, suggesting a limited impact. It may interact with membrane lipids, but not enough at low doses to cause rupture or significant damage (Serra et al., 2021). Lopes et al. (2014) reported that exposure to Gly at 5 and 10 mg/L did not significantly alter membrane structure in *D. rerio*, although slight reductions in fluidity were observed at 10 mg/L. In combined exposures, only treatments with 25 ppm Cd showed significant differences, suggesting that membrane damage is predominantly driven by Cd-induced oxidative stress, which leads to lipid peroxidation and disruption of membrane integrity. Cd exposure is well known to increase the generation of reactive oxygen species (ROS) and to overwhelm antioxidant defenses in fish tissues (Lee et al., 2023). Gly has also been shown to induce oxidative imbalance and disrupt antioxidant systems in fish (Faria et al., 2021), although generally with a milder cytotoxic profile. Hence, under combined exposure, the dominant membrane damage is likely due to Cd’s stronger oxidative insult, with Gly acting as a secondary stressor that modulates, but does not dictate, the overall cellular response.

Exposure to Cd, Gly, and their combination did not induce statistically significant differences in sperm DNA damage in *P*. *magdalenae* at any evaluated concentration. Although Cd is known to cause oxidative stress and DNA alterations in fish (Ali et al., 2022), the absence of damage in this study suggests the evaluated levels were not genotoxic. Similarly, studies on *D. rerio* have shown that Gly’s effects on DNA vary by formulation and exposure duration (Lopes et al., 2014). The lack of DNA damage may be related to compensatory mechanisms and antioxidant defenses, such as SOD and GPx, that neutralize ROS before they damage DNA (Gautam et al., 2024). Exposure to most combinations did not alter sperm DNA integrity; however, two specific treatments (Cd 0.0025 + Gly 40 mg/L and Cd 0.25 + Gly 40 mg/L) showed significant differences, which suggests that genotoxic effects may emerge under particular scenarios such as high Gly load in combination with low–moderate Cd. This pattern might be explained by compensatory antioxidant mechanisms being sufficient under many exposure regimes but overwhelmed in certain mixtures. Indeed, previous work in zebrafish demonstrated that Gly exposure reduces mitochondrial function, membrane integrity, and DNA integrity in sperm cells (Lopes et al., 2014). In addition, mixed exposures to Cd and Gly have been shown to produce synergistic DNA damage in tilapia tissues (Ibrahim et al., 2018). Further studies using oxidative stress biomarkers, longer exposures, and detoxification pathway analyses will help clarify the interactions of these compounds in fish reproductive systems.

Taken together, our results provide novel evidence of concentration-dependent interactions between Cd and Gly, emphasizing the importance of considering mixed effects when evaluating ecotoxicological risks in aquatic organisms. Although the concentrations tested in this study were higher than most values typically reported in natural environments, they still fall within ranges that could realistically occur during pollution peaks or in heavily impacted areas. Thus, our results may serve as an early warning of the biological risks linked to intensifying contamination, especially in regions under growing agricultural and mining pressure. A key limitation of this work is that it was conducted under controlled laboratory conditions, which cannot fully reproduce the complexity and variability of natural aquatic ecosystems. In the wild, fish are exposed to multiple stressors simultaneously and to fluctuating contaminant levels. Moreover, the absence of molecular or biochemical biomarkers such as oxidative stress indicators limits our ability to clarify the mechanisms driving the observed toxicity. Future research should therefore consider longer-term exposures, as suggested by Aguilar-Juárez et al. (2025), to strengthen these findings and provide more robust insights for ecological risk assessment.

## 5 Conclusion

This study provides new evidence on how combined exposure to Cd and Gly affects fish sperm quality, revealing complex and concentration-dependent interactions between these contaminants. Our results demonstrate that co-exposure poses a greater risk to sperm viability than either compound alone. A toxicity threshold was identified beyond which Gly impairs sperm motility, while Cd primarily drives membrane damage through oxidative stress. The interaction between both compounds exhibits both antagonistic and synergistic patterns depending on concentration ratios, suggesting the involvement of compensatory antioxidant mechanisms that may mitigate damage under certain conditions but fail under specific mixtures. Overall, these findings advance the understanding of Cd–Gly co-exposure effects on fish reproduction and highlight the need for longer-term *in situ* studies incorporating biomarker-based approaches to better characterize ecological risk.

## Data Availability

The raw data supporting the conclusions of this article will be made available by the authors, without undue reservation.
