# Supplementary figures and images for: Cadmium and glyphosate jointly compromise sperm function, fertilization, and early development in Prochilodus magdalenae
Source: Front Toxicol. 2025 Nov 24;7:1698489. doi: 10.3389/ftox.2025.1698489 (PMC12682675; doi:10.3389/ftox.2025.1698489)

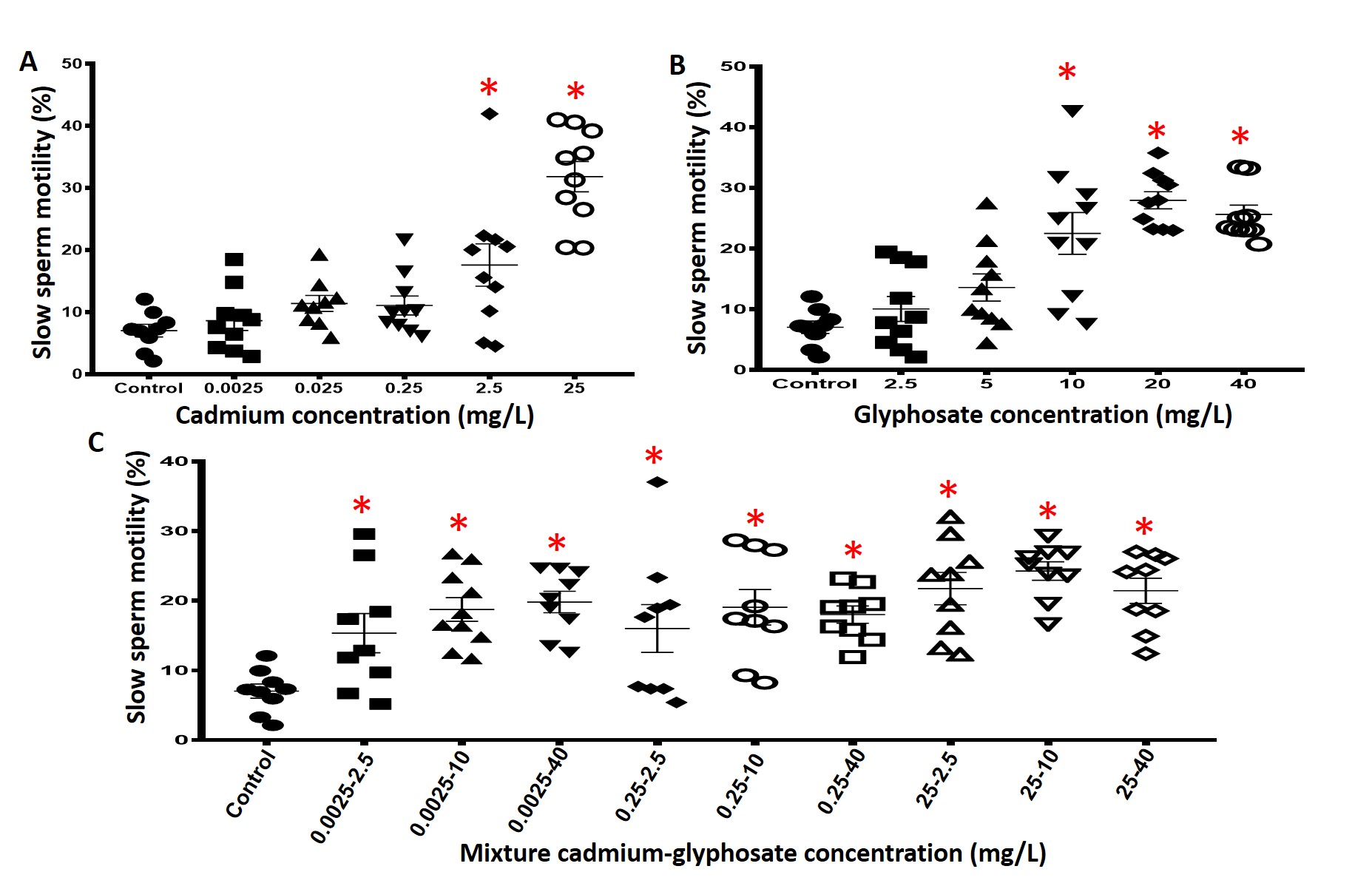

Supplement: Supplementary file 1 [file Image3.jpeg]

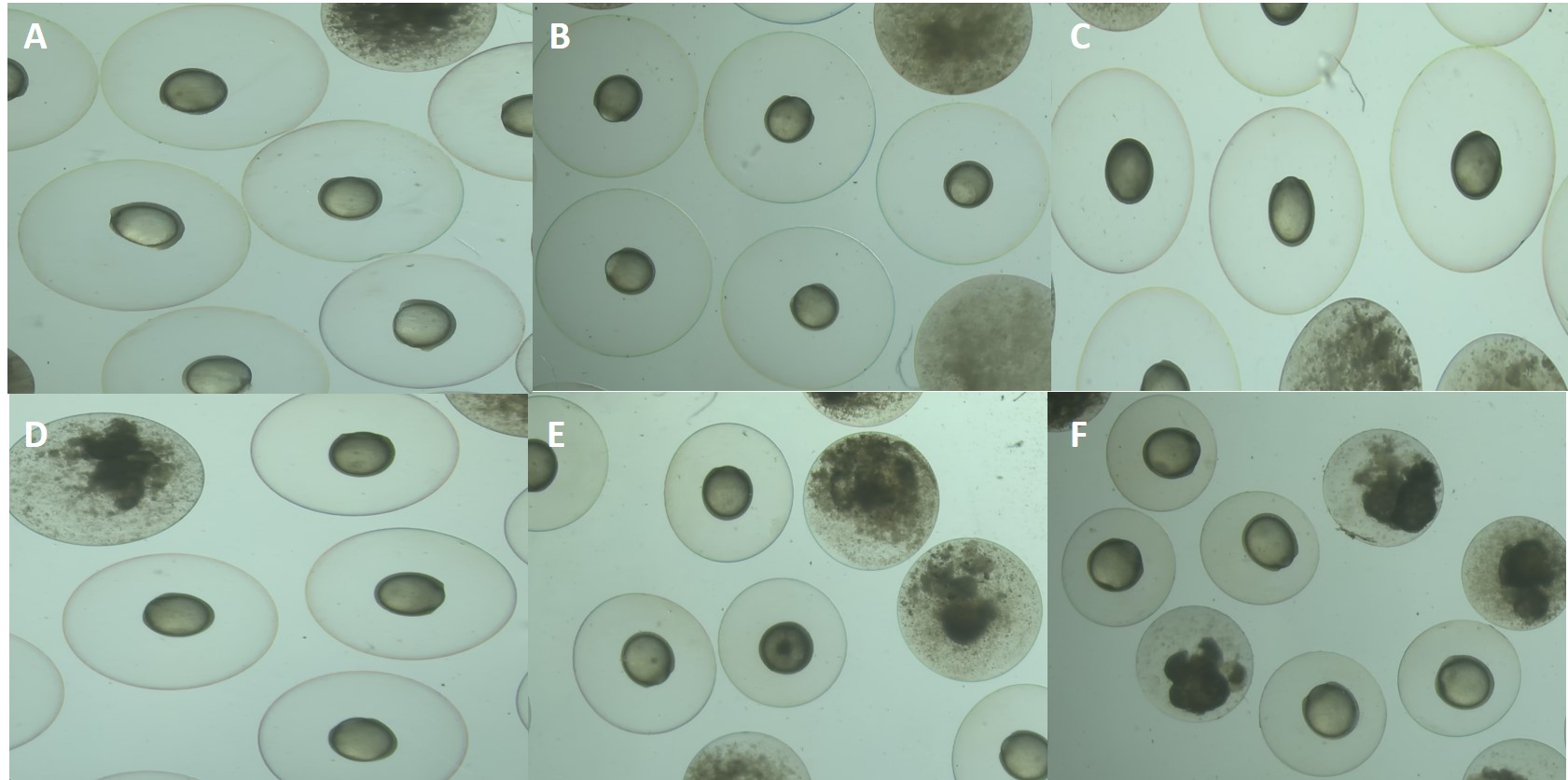

Supplement: Supplementary file 3 [file Image9.jpeg]

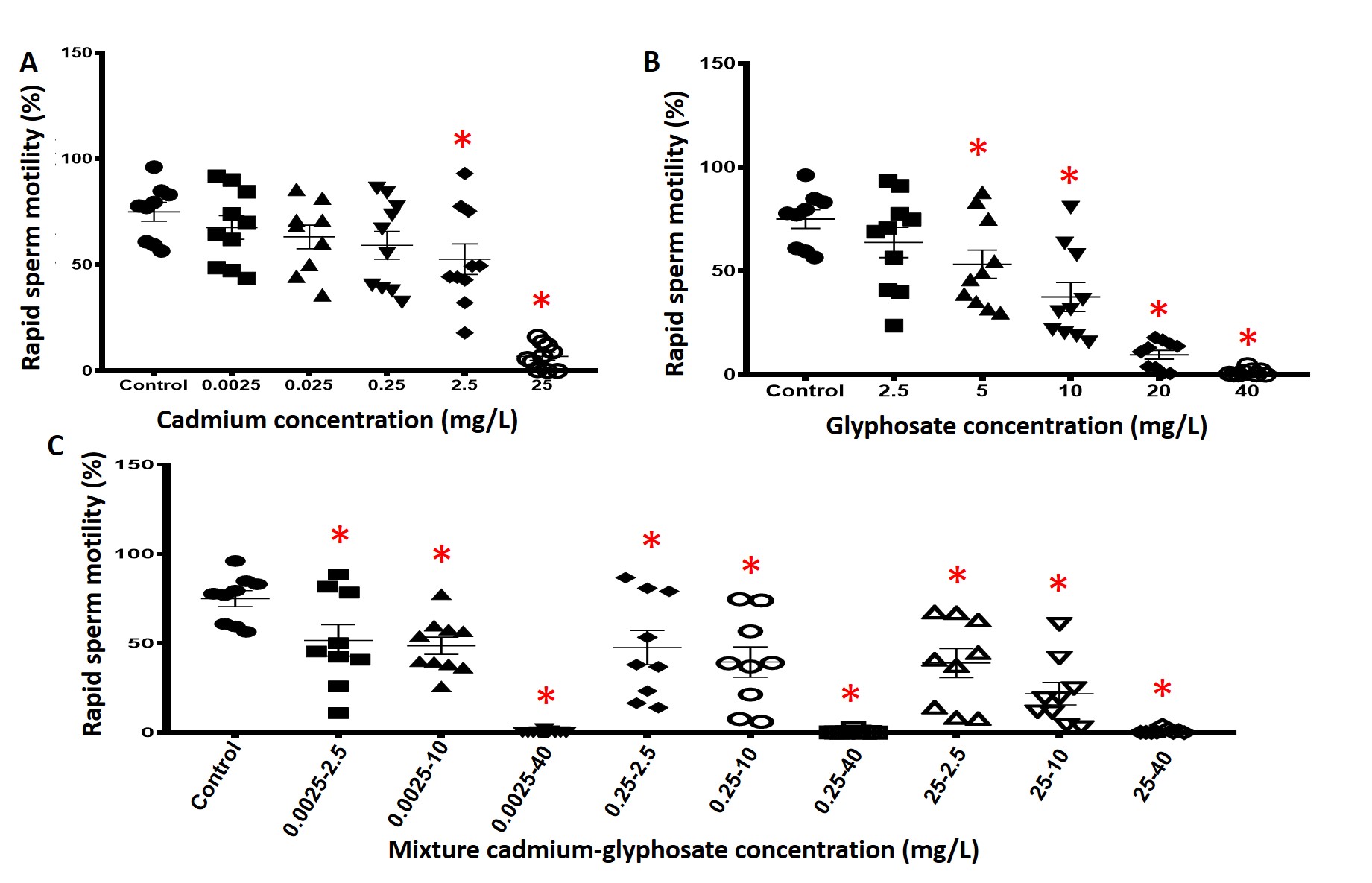

Supplement: Supplementary file 4 [file Image1.jpeg]

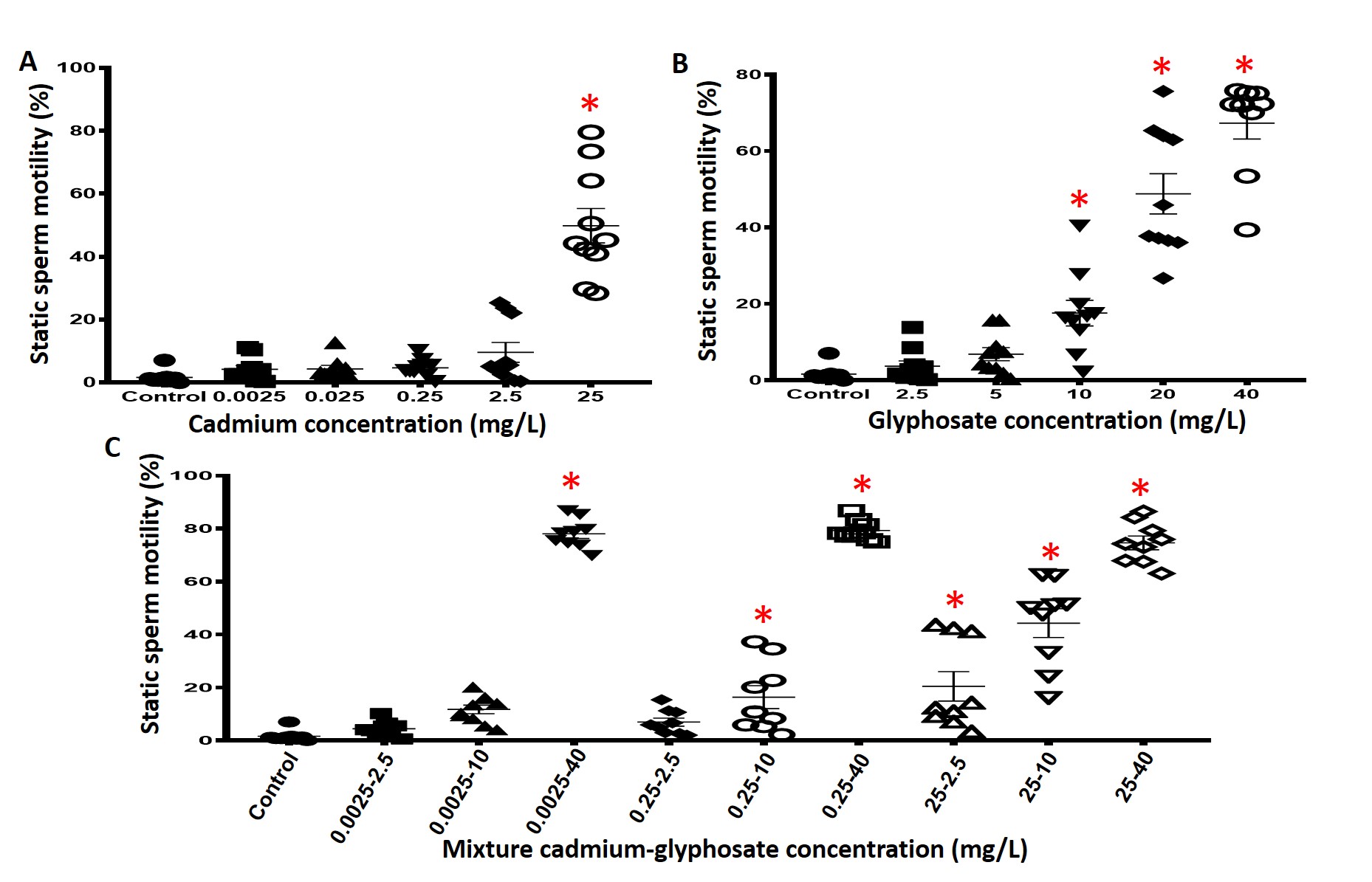

Supplement: Supplementary file 5 [file Image4.jpeg]

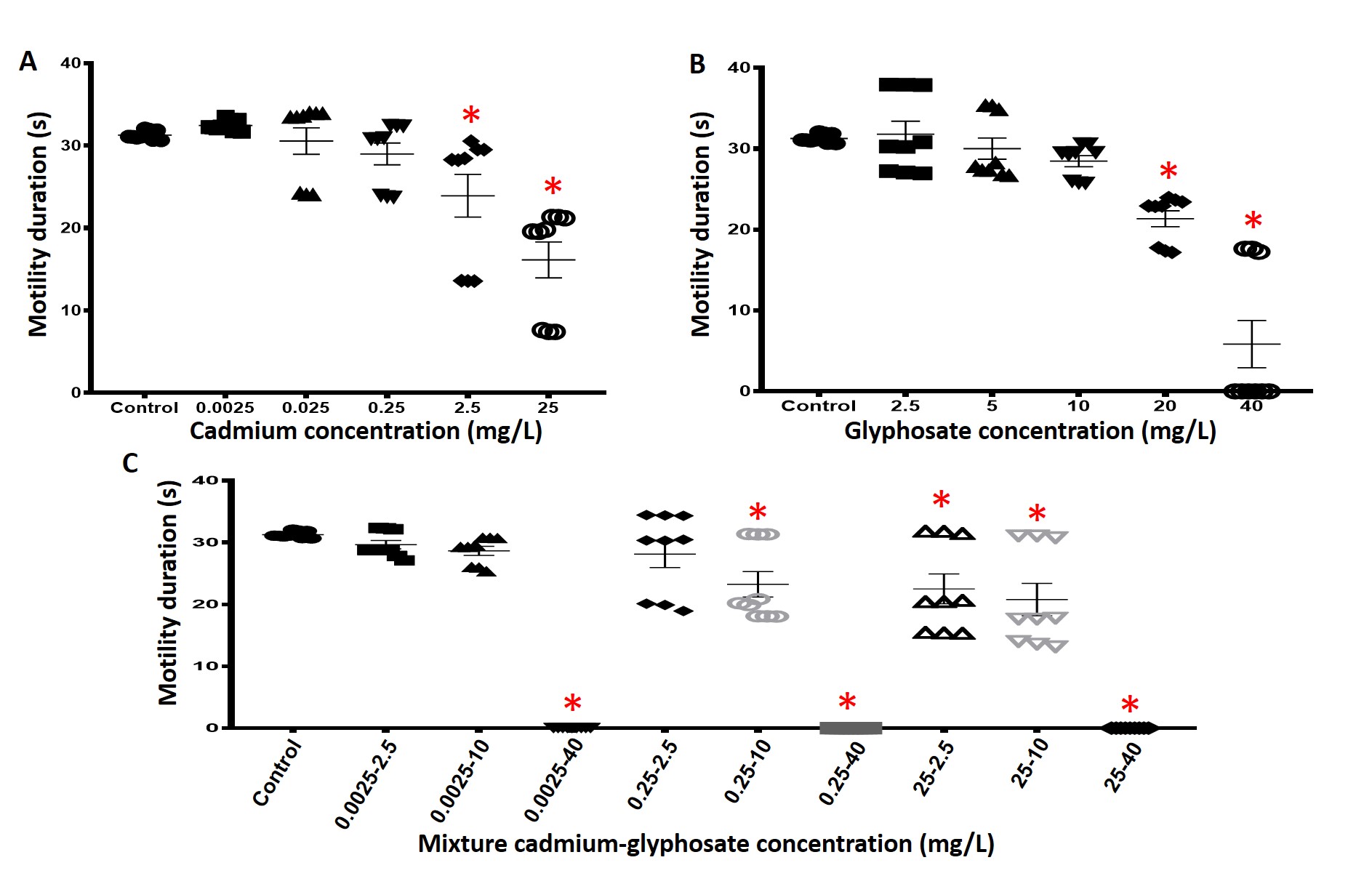

Supplement: Supplementary file 6 [file Image7.jpeg]

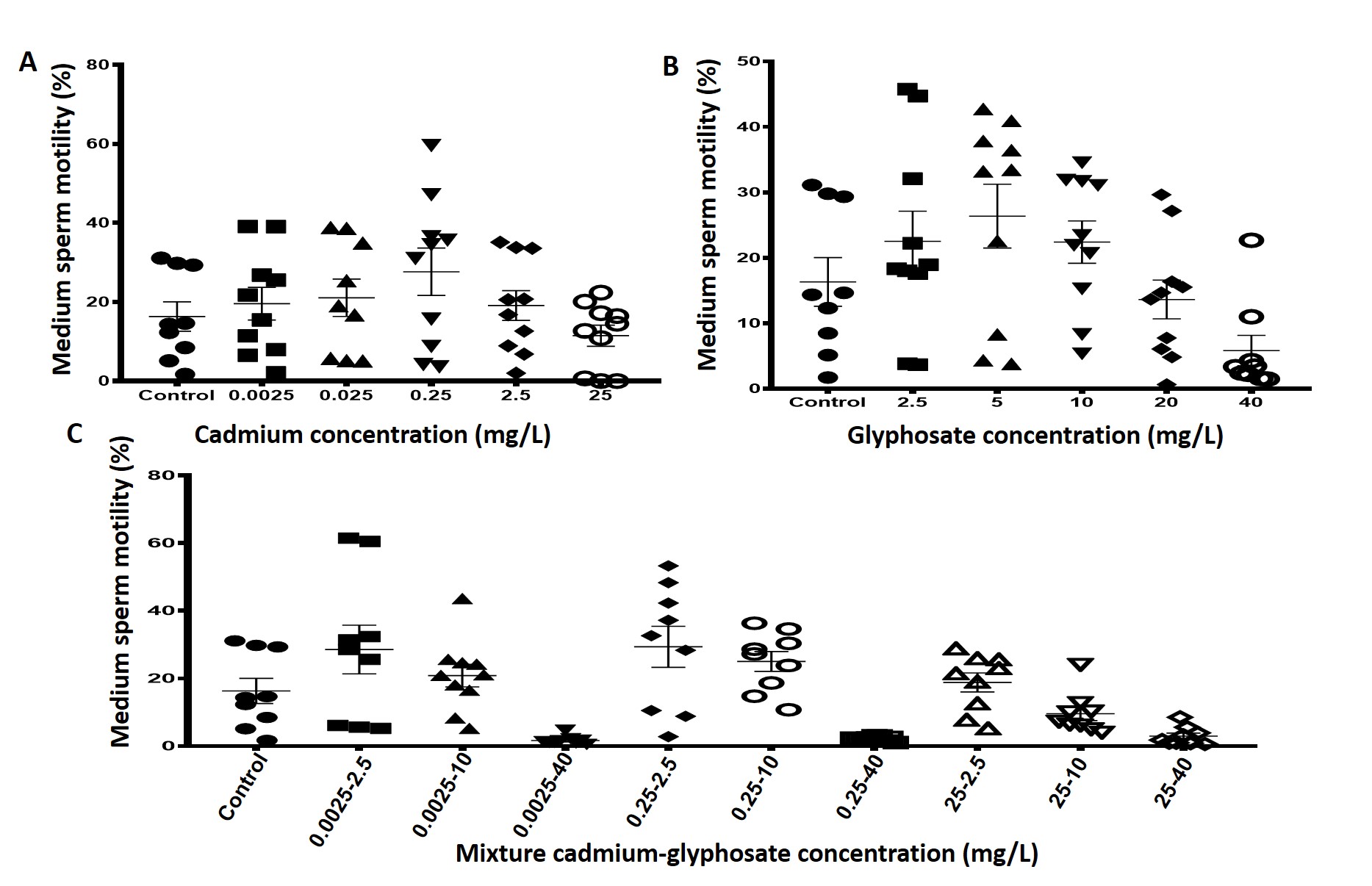

Supplement: Supplementary file 7 [file Image2.jpeg]

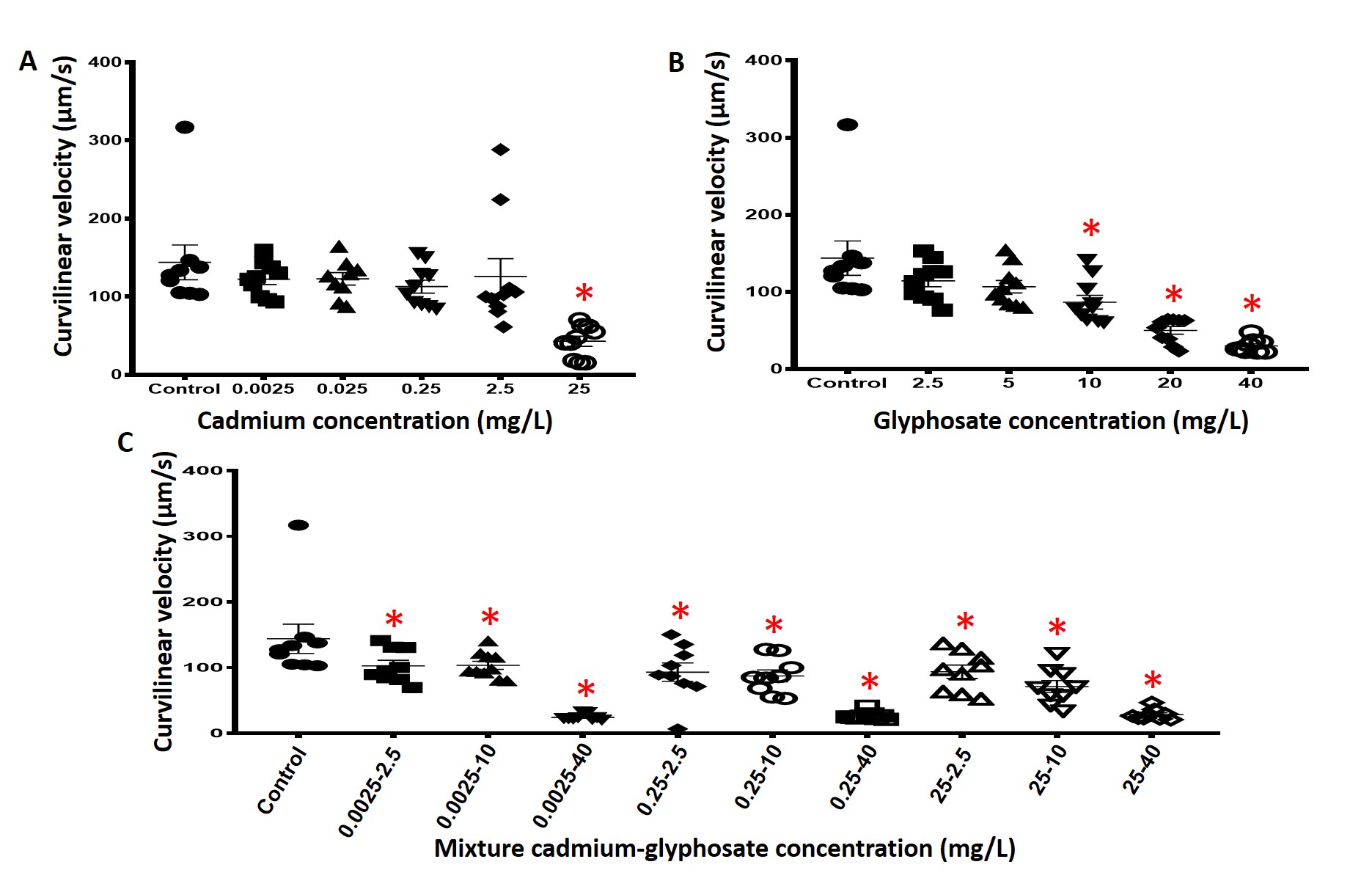

Supplement: Supplementary file 8 [file Image5.jpeg]

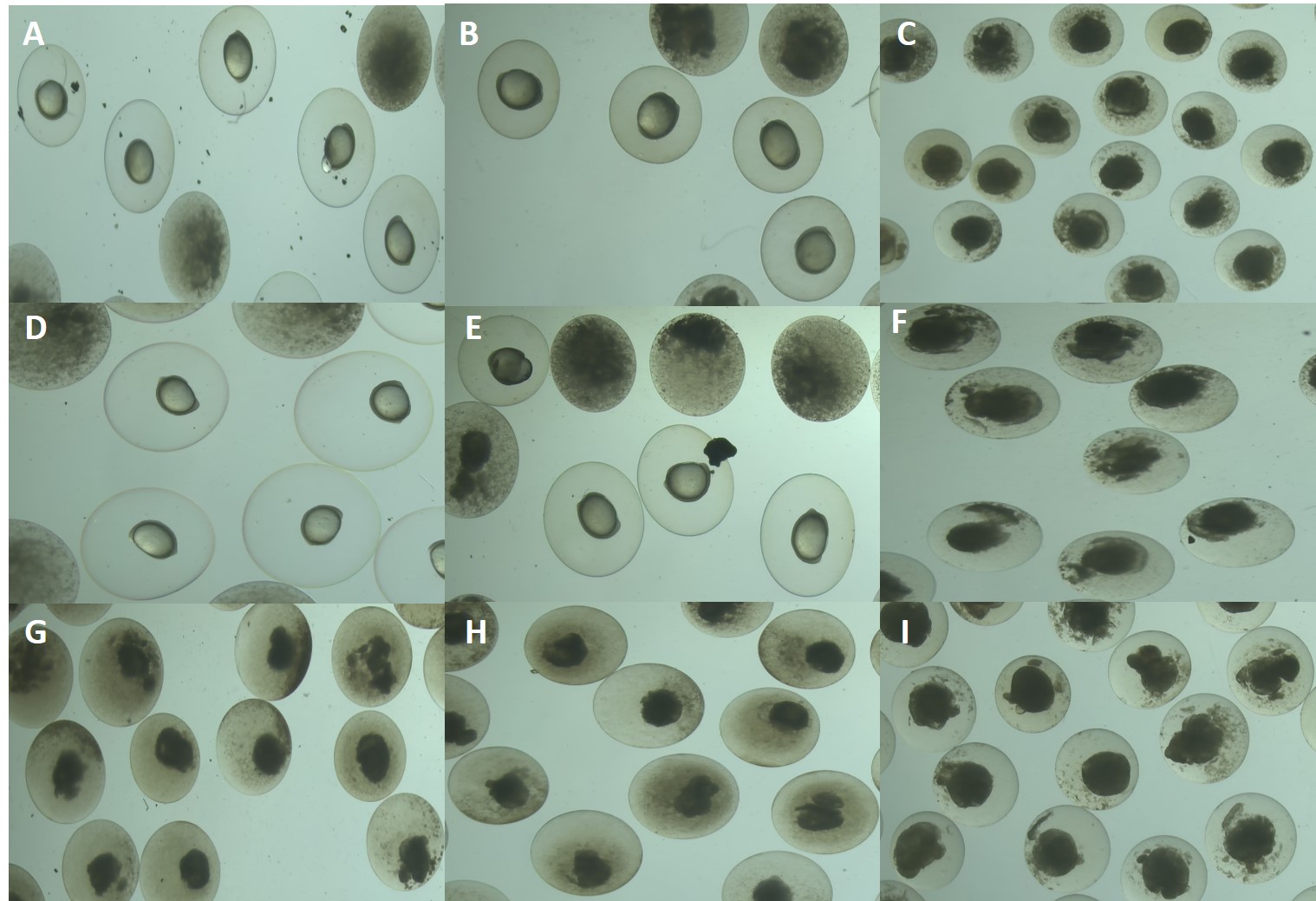

Supplement: Supplementary file 9 [file Image10.jpeg]

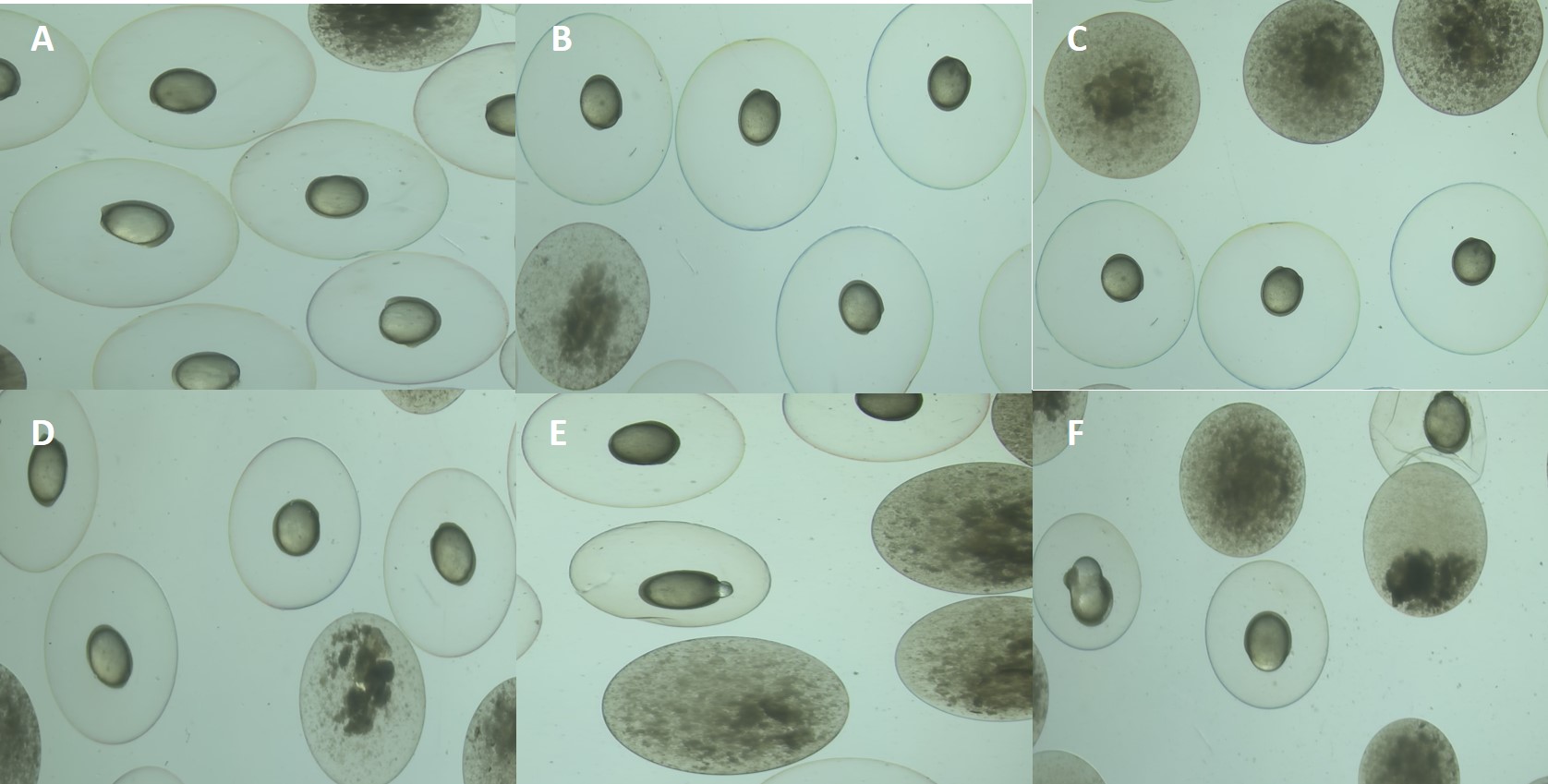

Supplement: Supplementary file 10 [file Image8.jpeg]

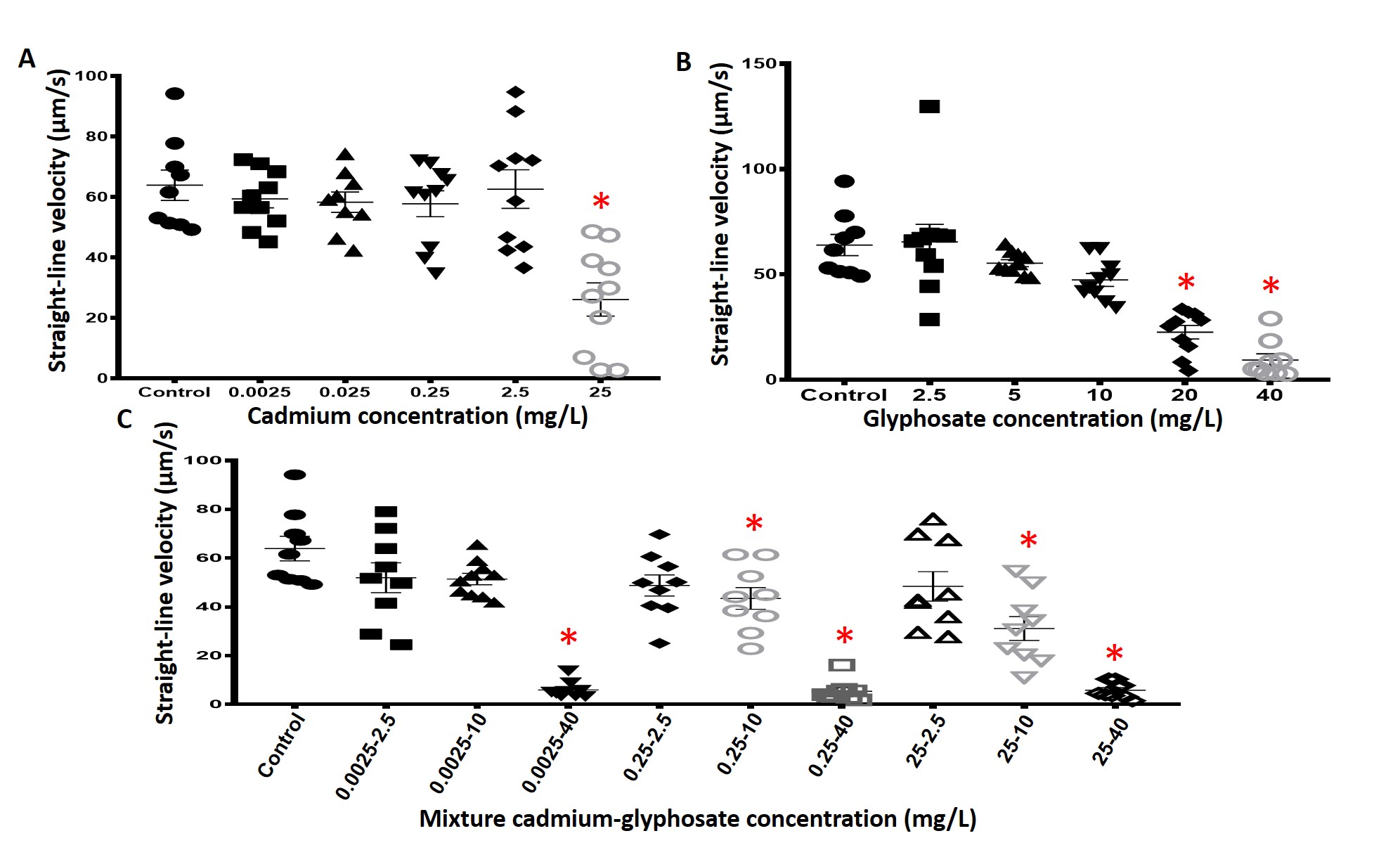

Supplement: Supplementary file 11 [file Image6.jpeg]
