# Supplementary material for: Cadmium and glyphosate jointly compromise sperm function, fertilization, and early development in Prochilodus magdalenae
Source: Front Toxicol. 2025 Nov 24;7:1698489. doi: 10.3389/ftox.2025.1698489 (PMC12682675; doi:10.3389/ftox.2025.1698489)
Supplement: Supplementary file 2 [file Table1.docx]

**Supplementary Figures**

**SF1.** Percentage of rapid spermatozoa of *P. magdalenae* semen exposed to Cd (A), Gly (B), or their combination (C). Values are expressed as mean ± standard error (SE). Asterisks indicate significant differences compared to the control group (p < 0.05).

**SF2.** Percentage of medium spermatozoa of *P. magdalenae* semen exposed to Cd (A), Gly (B), or their combination (C). Values are expressed as mean ± standard error (SE). Asterisks indicate significant differences compared to the control group (p < 0.05).

**SF3.** Percentage of slow spermatozoa of *P. magdalenae* semen exposed to Cd (A), Gly (B), or their combination (C). Values are expressed as mean ± standard error (SE). Asterisks indicate significant differences compared to the control group (p < 0.05).

**SF4.** Percentage of static spermatozoa of *P. magdalenae* semen exposed to Cd (A), Gly (B), or their combination (C). Values are expressed as mean ± standard error (SE). Asterisks indicate significant differences compared to the control group (p < 0.05).

**SF5.** Curvilinear velocity (VCL, µm/s) of *P. magdalenae* spermatozoa exposed to Cd (A), Gly (B), or their combination (C). Values are expressed as mean ± standard error (SE). Asterisks indicate significant differences compared to the control group (p < 0.05).

**SF6.** Straight-line velocity (VSL, µm/s) of *P. magdalenae* spermatozoa exposed to Cd (A), Gly (B), or their combination (C). Values are expressed as mean ± standard error (SE). Asterisks indicate significant differences compared to the control group (p < 0.05).

**SF7.** Motility duration (s) of *P. magdalenae* spermatozoa exposed to Cd (A), Gly (B), or their combination (C). Values are expressed as mean ± standard error (SE). Asterisks indicate significant differences compared to the control group (p < 0.05)

**SF8.** Embryos of *P. magdalenae* exposed to increasing concentrations of cadmium (Cd) at 6 hours post-fertilization (hpf). (A) Control, (B) 0.0025 mg/L, (C) 0.025 mg/L, (D) 0.25 mg/L, (E) 2.5 mg/L, and (F) 25 mg/L Cd.

**SF9.** Embryos of *P. magdalenae* exposed to increasing concentrations of glyphosate (Gly) at 6 hours post-fertilization (hpf). (A) Control, (B) 2.5 mg/L, (C) 5 mg/L, (D) 10 mg/L, (E) 20 mg/L, and (F) 40 mg/L Gly.

**SF10.** Embryos of *P. magdalenae* exposed to mixtures of cadmium (Cd) and glyphosate (Gly) at 6 hours post-fertilization (hpf). (A) Cd 0.0025 + Gly 2.5 mg/L, (B) Cd 0.0025 + Gly 10 mg/L, (C) Cd 0.0025 + Gly 40 mg/L, (D) Cd 0.25 + Gly 2.5 mg/L, (E) Cd 0.25 + Gly 10 mg/L, (F) Cd 0.25 + Gly 40 mg/L, (G) Cd 25 + Gly 2.5 mg/L, (H) Cd 25 + Gly 10 mg/L, and (I) Cd 25 + Gly 40 mg/L.
